# Sensitivity of human pluripotent stem cells to insulin precipitation induced by peristaltic pump-based medium circulation: considerations on process development

**DOI:** 10.1038/s41598-017-04158-x

**Published:** 2017-06-21

**Authors:** Diana Massai, Emiliano Bolesani, Diana Robles Diaz, Christina Kropp, Henning Kempf, Caroline Halloin, Ulrich Martin, Tudor Braniste, Giuseppe Isu, Vanessa Harms, Umberto Morbiducci, Gerald Dräger, Robert Zweigerdt

**Affiliations:** 1 Leibniz Research Laboratories for Biotechnology and Artificial Organs (LEBAO), Department of Cardiac, Thoracic, Transplantation and Vascular Surgery, Hannover Medical School, Carl-Neuberg-Str. 1, 30625 Hannover, Germany; 2 REBIRTH-Cluster of Excellence, Hannover Medical School, Carl-Neuberg-Str. 1, 30625 Hannover, Germany; 3 National Center for Materials Study and Testing, Technical University of Moldova, Bv. Stefan cel Mare 168, Chisinau, 2004 Republic of Moldova; 4 Department of Mechanical and Aerospace Engineering, Politecnico di Torino, Corso Duca degli Abruzzi 24, 10129 Torino, Italy; 5 Department of Biomedicine, University of Basel and Department of Surgery, University Hospital of Basel, 4031 Basel, Switzerland; 6 Institute of Organic Chemistry, Leibniz University Hannover, Schneiderberg 1B, 30167 Hannover, Germany

## Abstract

Controlled large-scale production of human pluripotent stem cells (hPSCs) is indispensable for their envisioned clinical translation. Aiming at advanced process development in suspension culture, the sensitivity of hPSC media to continuous peristaltic pump-based circulation, a well-established technology extensively used in hydraulically-driven bioreactors, was investigated. Unexpectedly, conditioning of low protein media (*i.e*. E8 and TeSR-E8) in a peristaltic pump circuit induced severe viability loss of hPSCs cultured as aggregates in suspension. Optical, biochemical, and cytological analyses of the media revealed that the applied circulation mode resulted in the reduction of the growth hormone insulin by precipitation of micro-sized particles. Notably, in contrast to insulin depletion, individual withdrawal of other medium protein components (*i.e*. bFGF, TGFβ1 or transferrin) provoked minor reduction of hPSC viability, if any. Supplementation of the surfactant glycerol or the use of the insulin analogue Aspart did not overcome the issue of insulin precipitation. In contrast, the presence of bovine or human serum albumin (BSA or HSA, respectively) stabilized insulin rescuing its content, possibly by acting as molecular chaperone-like protein, ultimately supporting hPSC maintenance. This study highlights the potential and the requirement of media optimization for automated hPSC processing and has broad implications on media development and bioreactor-based technologies.

## Introduction

Human pluripotent stem cells (hPSCs), due to their essentially unlimited proliferative capacity and their potential to differentiate into any somatic cell type *in vitro*, represent a superior cell source for biomedical sciences^1, 2^. In the perspective of their clinical and industrial applications, it was recently shown that hPSCs, including human embryonic (hESCs) and human induced pluripotent (hiPSCs) cells, can be cultivated as “matrix-free cell only aggregates” in static and dynamic suspension culture^3–5^. This approach eliminates matrix requirements, enables transition from surface-dependent two-dimensional (2D) culture towards three-dimensional (3D) cultivation, and supports development and up-scaling of good manufacturing practice (GMP)-compliant processes^2, 6–9^. However, recent studies revealed that the 2D-to-3D switch induces distinct molecular changes in hPSCs such as the differential cleavage of E-cadherin, thereby modulating cell-cell communication and the activity of specific pathways, particularly WNT signaling^10^. Moreover, high sensitivity of hPSCs to physicochemical culture parameters in suspension has been demonstrated^6, 11, 12^. For instance, turbulent hydrodynamic conditions and subsequent shear stresses in stirred culture vessels can markedly affect hPSC aggregates by reducing cell viability ﻿and disrupting the sensitive equilibrium of pluripotency versus differentiation, and can lead to undesired culture heterogeneity^9, 13, 14^. Thus, for the successful hPSC processing in bioreactors, several parameters including bioreactor design (*e.g*. vessel shape, dimensions, geometry and the arrangement of impellers, probes, ports, *etc*.) and working conditions (*e.g*. agitation speed, working volume, *etc*.) must be considered^9, 13, 15, 16^. Further process-determining factors comprise culture media composition and stability^17, 18^. Since their initial use for hESC culture^19^, growth media for hPSC culture have undergone a continuous evolution with the ultimate goal to develop serum-free, xeno-free, and chemically defined formulations suitable for therapeutic application^18^. In addition, the complex interplay of environmental parameters, namely dissolved oxygen concentration (DO), pH, cell density, cell feeding strategies, and others, should be considered^6, 20, 21^.

Consequently, increasing efforts were dedicated to the development of universal, controlled and predictive hPSC mass production strategies^22^, particularly in industry-compliant instrumented stirred-tank bioreactors^6, 8, 9, 18, 23, 24^. Indeed, applying “matrix-free cell only aggregate” culture in the complex medium mTeSR1^15^ or in the chemically defined xeno-free medium E8^17^ in combination with single-use stirred-tank bioreactors^21^ led to encouraging progress^9, 24^.

However, linear - rather than aspired exponential - growth kinetics of hPSCs were achieved in impeller stirred-tank bioreactors^15, 21^. Additionally, aggregate size heterogeneity, which might impact on subsequent differentiation results^25, 26^, was described in several stirred culture platforms^5, 9, 13, 14, 24^. This suggests that numerous limitations still exist, triggering investigations into alternative culture technologies.

Recently, an impeller-free bioreactor for dynamic suspension culture was proposed^27^. The system exploits laminar hydrodynamics. Thanks to an appositely designed vessel geometry and the connection to a circulation circuit, buoyant vortices form within the culture chamber allowing homogeneous distribution of cells/aggregates in suspension at low-shear conditions^27^. Medium flow in this system is achieved by peristaltic pump technology^28^, which is extensively used in a broad range of hydraulically driven bioreactors requiring continuous medium circulation^29–32^.

In order to test the applicability of this platform for suspension culture of hPSCs, this study investigated how specific hPSC expansion media (*i.e*. E8, TeSR-E8, mTeSR1 and StemMACS iPS-Brew XF) comply with continuous peristaltic pump-based circulation. The findings revealed an unexpected sensitivity of specific media components to the applied circulation mode. In particular, in low protein media E8 and TeSR-E8, peristaltic pumping induced physical instability of the growth hormone insulin which precipitated in insoluble particles. Relevant bioassays, based on hiPSC and hESC lines, revealed unreported sensitivity of hPSCs to reduced insulin concentrations. In contrast, sequential omission of other factors that are known to be essential for maintaining hPSC pluripotency, including bFGF or TGFβ1^17, 18^, showed minor short term effects on cell viability and the integrity of hPSC aggregates. Notably, in the presence of bovine or human serum albumin (BSA or HSA, respectively), detrimental effects on hPSCs were limited despite the peristaltic pump conditioning of respective media, suggesting that albumin stabilizes dissolved insulin in hPSC media. While the physical instability of insulin in solution is well known in the diabetes research and industrial field^33–35^, inadequate attention to this subject has been dedicated in hPSC culture media development.

Due to the necessity of the hPSC field to move towards GMP-compliant, chemically defined low protein media compositions^2, 9, 36^ and the extensive use of peristaltic pumps in bioprocessing^37^, this study has substantial impact within and beyond hPSC manufacturing.

## Results

### Disruption of hPSC aggregate culture by peristaltic pump conditioned E8 was mediated by insulin depletion

**Figure 1:**
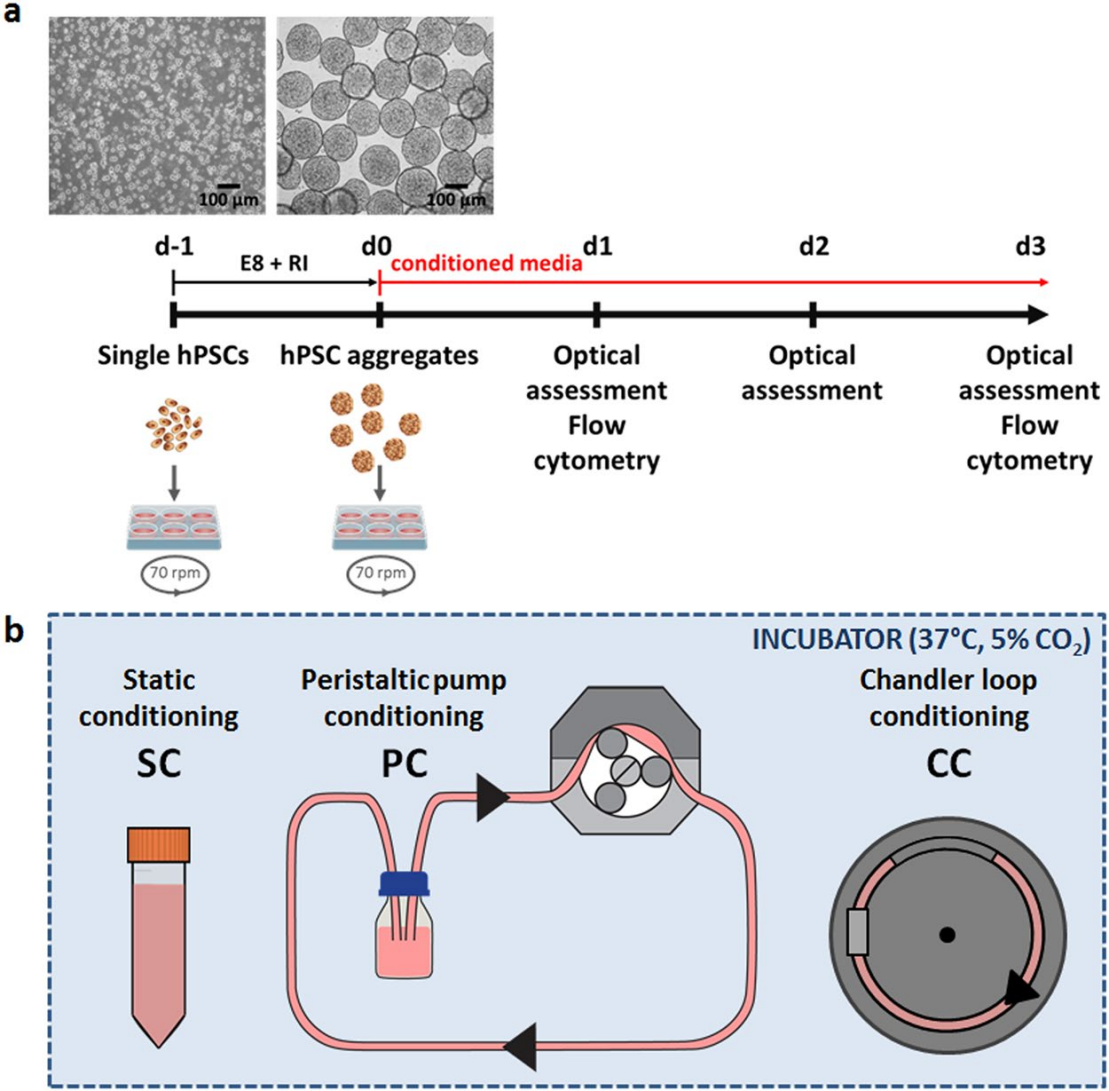
hPSC aggregate suspension culture and hPSC culture media conditioning. (**a**) Schematic of hPSC aggregate culture set-up including representative light microscopy pictures. Single hPSCs were inoculated (d-1) and positioned for aggregate formation on an orbital shaker for 24 h. hPSC aggregates formed at d0 were harvested and cultured with conditioned media on an orbital shaker for further 72 h. Optical assessment was performed daily, with flow cytometry analysis on d1 and d3. (**b**) Schematic of hPSC culture media conditioning set-up including static conditioning in 50 mL tube (SC), continuous circulation within a closed-loop peristaltic pump-based circuit (PC), and continuous circulation within a chandler loop system (CC).

**Figure 2:**
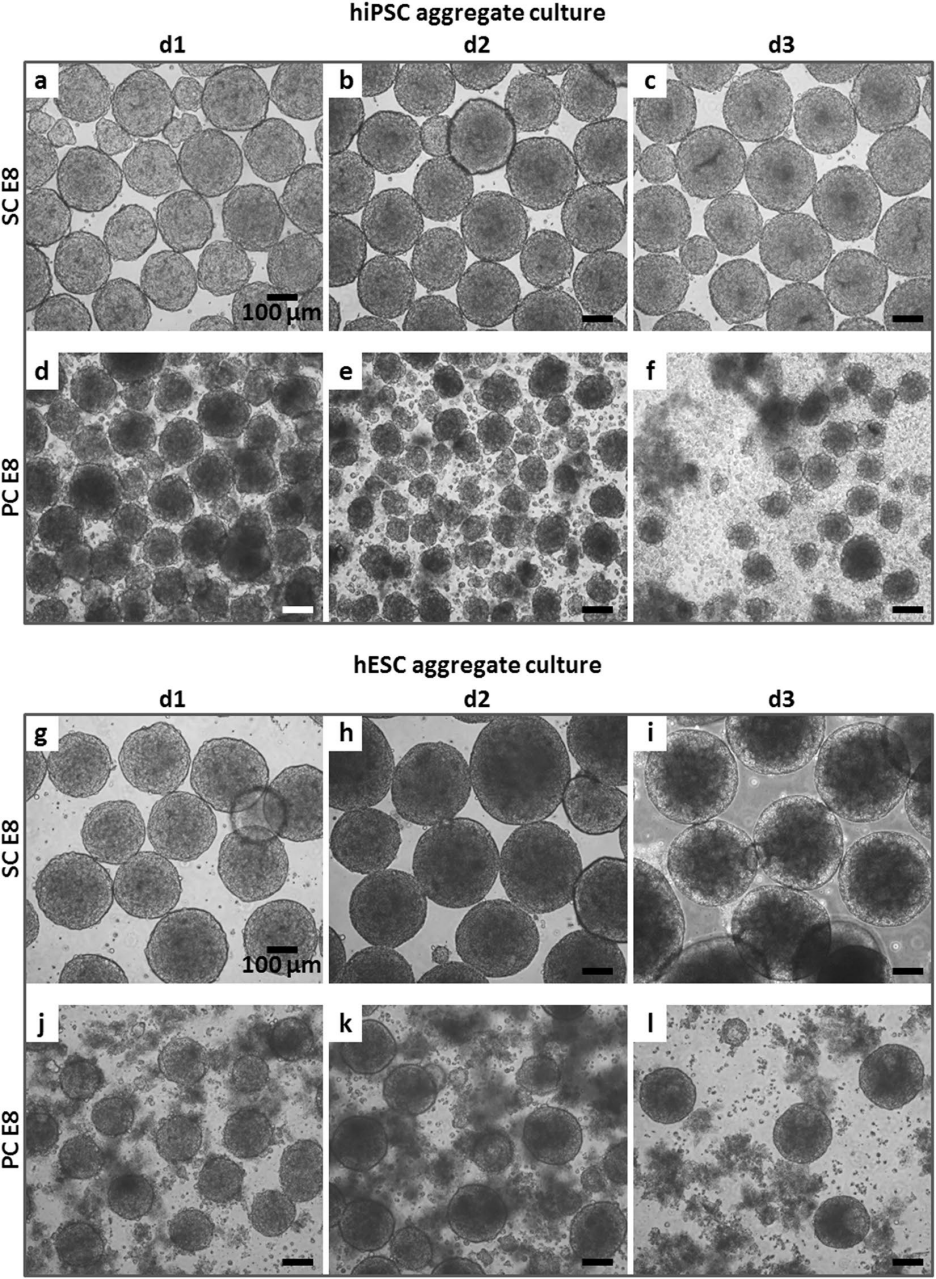
hPSC aggregates cultured in suspension with SC E8 and PC E8. Light microscopy images showed that (**a–c**) hiPSC and (**g–i**) hESC aggregates cultured in SC E8 maintained their spherical morphology and increased in diameter over the 3 days. In contrast, light microscopy images of (**d**–**f**) hiPSC and (**j**–**l**) hESC aggregates cultured in PC E8 showed floating single cells and aggregates with irregular morphology already on d1, followed by an increase in cell debris and disaggregation on d2–3. Scale bar = 100 µm.

Respective hiPSC and hESC aggregates pre-formed by established, single cell-inoculated suspension (Fig. 1a)^3, 5^ were cultured in hPSC media conditioned by treatments schematically depicted in Fig. 1b. In static conditioned E8 medium (SC E8), hPSC aggregates maintained their spherical morphology and increased in diameter over the 3 days assessed (Fig. 2a–c for hiPSCs and Fig. 2g–i for hESCs), according to published data^3–5^. Unexpectedly, light microscopy images of hPSC aggregates cultured in peristaltic pump conditioned E8 medium (PC E8) showed floating single cells and hPSC aggregates with irregular morphology and reduced size compared to the respective SC E8 controls already after one day of cultivation, followed by increase in cell debris and disaggregation on days 2–3 (Fig. 2d–f for hiPSCs and Fig. 2j–l for hESCs).

In search for the underlying mechanism, E8 medium was individually conditioned with all physical components of the peristaltic circuit in direct contact with the medium (*i.e*. tubing, connections, and the medium reservoir). None of these conditioned media induced detrimental effects on hPSC aggregates (data not shown), confirming cytocompatibility of all components used in the system.

**Figure 3:**
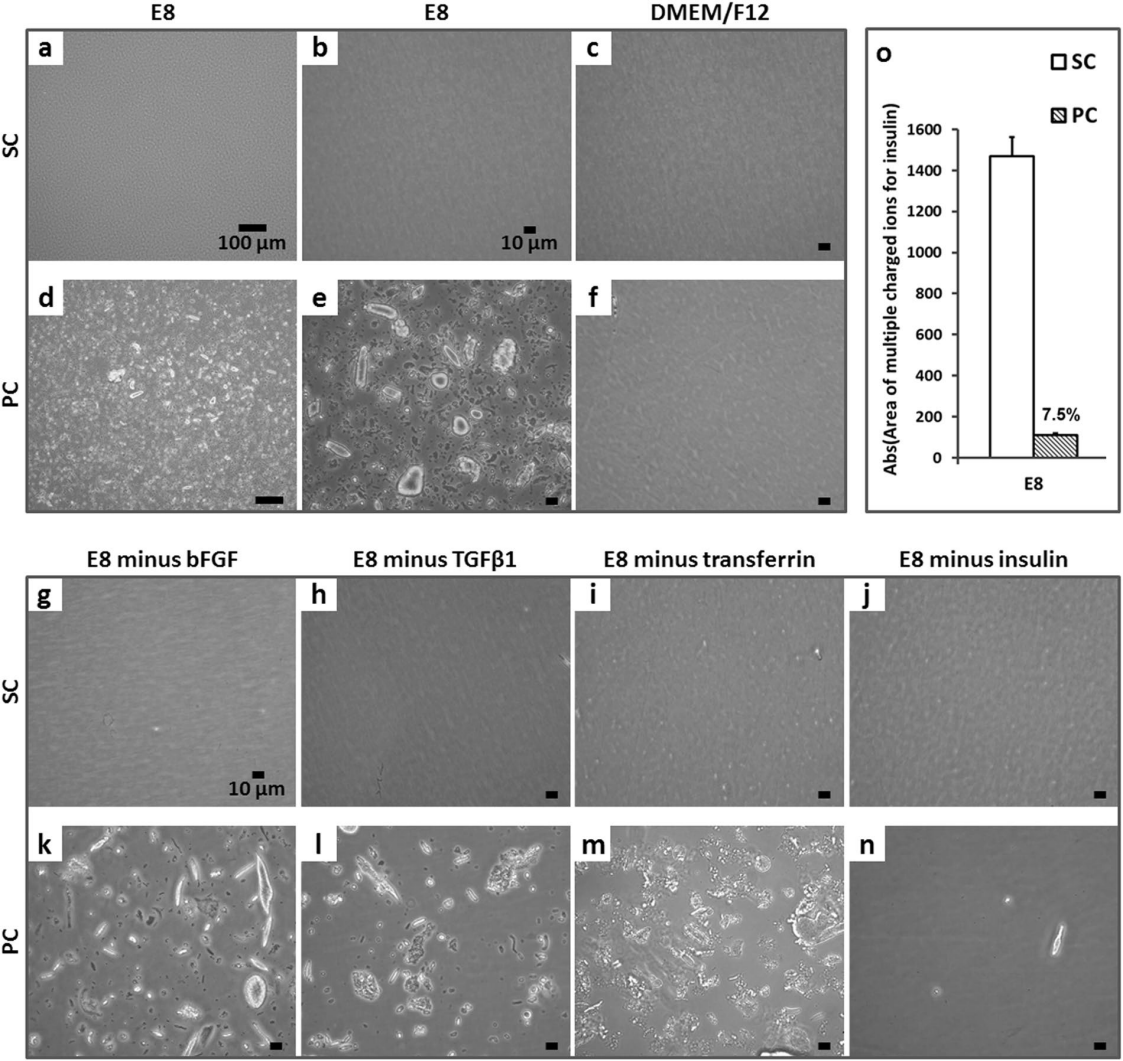
Optical inspection of conditioned media. Light microscopy images of SC E8 at (**a**) 10x (scale bar = 100 µm) and (**b**) 40x magnification (scale bar = 10 µm) revealed a transparent medium, whereas in (**d–e**) PC E8 the presence of irregular particles of up to ~50 µm was observed. The (**c**) static and (**f**) peristaltic pump conditioning of DMEM/F12 did not indicate any release of particles from circuit components (scale bar = 10 µm). Similarly, (**g**–**j**) no particles were detectable in any SC E8 media lacking one protein at a time omitted before conditioning (scale bar = 10 µm). In contrast, peristaltic pump conditioning resulted in substantial particle formation in (**k**) E8 minus bFGF, (**l**) E8 minus TGFβ1, and (**m**) E8 minus transferrin, except in (**n**) E8 minus insulin (scale bar = 10 µm). (**o**) Semi-quantitative UPLC-MS analysis of SC E8 and PC E8 media revealed that less than 10% of dissolved insulin remained in PC E8 compared to SC E8 controls (n = 2).

Notably, light microscopy of SC E8 (Fig. 3a and b) and PC E8 (Fig. 3d and e) revealed the presence of irregular particles of up to ~50 µm in size in PC E8, only. Extensive sterility tests excluded microbial contamination of any native or conditioned medium tested throughout the study (data not shown). To investigate the potential presence of dust or release of debris and degradation material, *e.g*. due to spallation of the tubing, deionized water or DMEM/F12 (Fig. 3c), the basic medium for E8 lacking any supplementation of E8-specific proteins, were conditioned within the peristaltic circuit. This analysis did not indicate any release of particles from the applied circuit components (Fig. 3f), rather it suggested a relationship between protein components in E8 and the formation of the aforementioned particles in combination with peristaltic pump conditioning.

To test this hypothesis, E8 lacking one protein at a time (*i.e*. E8 minus bFGF, -TGFβ1, -transferrin, -insulin) was conditioned by static and peristaltic pump treatment, respectively. Light microscopy revealed that no particles were detectable in any SC medium (Fig. 3g–j), as expected. In contrast, applying peristaltic pump conditioning resulted in substantial particle formation in all media (Fig. 3k–m), except in the absence of insulin (Fig. 3n).

Moreover, semi-quantitative UPLC-MS analysis of SC E8 and PC E8 media samples revealed that less than 10% of dissolved insulin was present in PC E8 compared to SC E8 controls (Fig. 3o). Together, this strongly suggested that the insulin in E8 was underlying the formation of particle-like precipitates induced by peristaltic pump-based circulation, as confirmed by particle solubilization (Supplementary Figures S1 and S2). In consequence, dissolved insulin was almost entirely depleted within 12 h.

**Figure 4:**
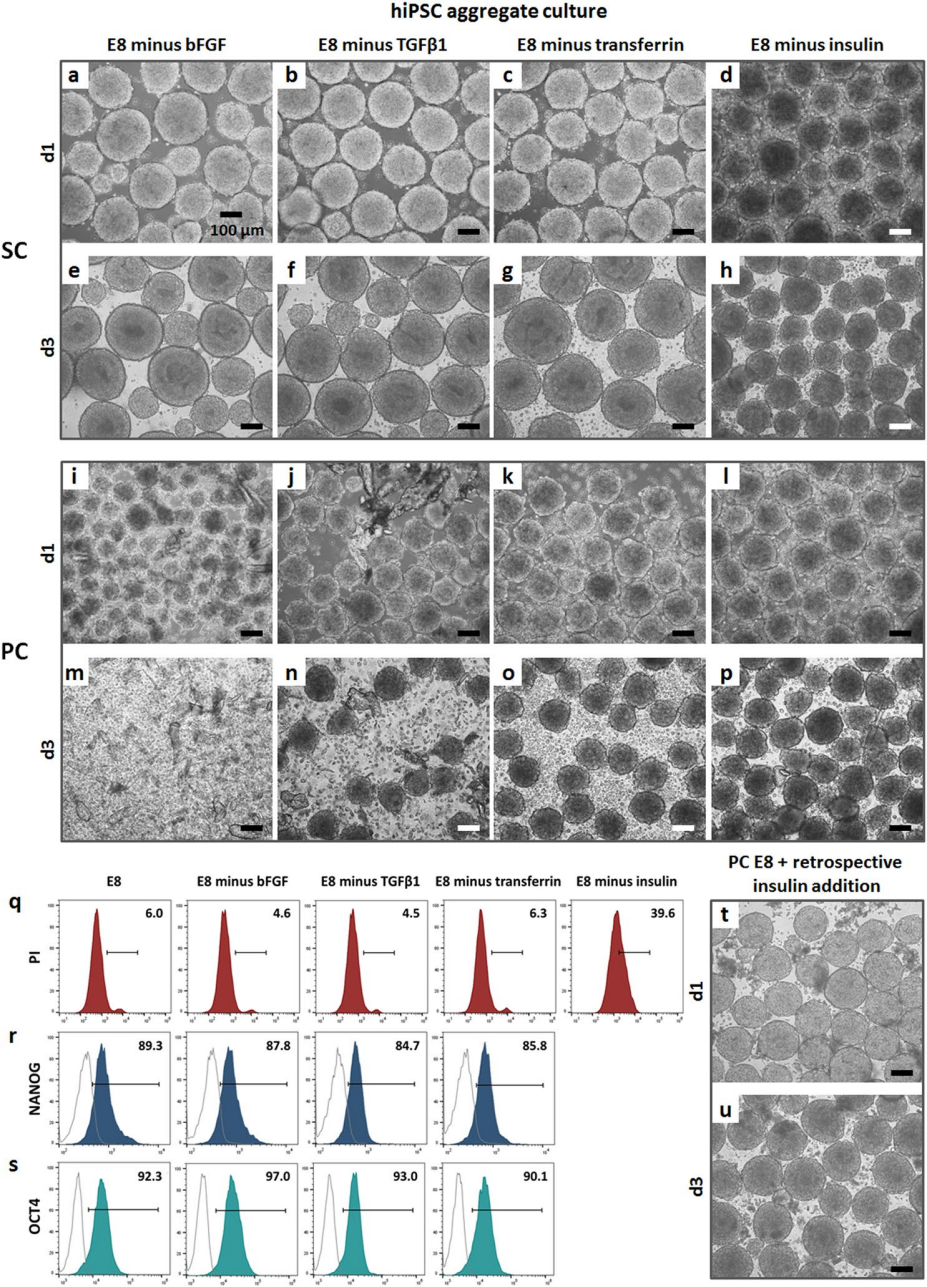
hiPSC aggregates cultured in suspension with SC E8 and PC E8 lacking one protein at a time omitted before conditioning. Light microscopy images of hiPSC aggregates, on day 1 and day 3, cultured in SC E8 lacking one protein at a time, omitted before conditioning, showed that omission of either (**a,e**) bFGF, (**b,f**) TGFβ1 or (**c,g**) transferrin had only minor effects on hiPSC aggregate morphology. In contrast, omitting insulin resulted in (**d**) apparent shading of hiPSC aggregates readily after 24 h and (**h**) progressive shrinkage thereafter. Light microscopy images of hiPSC aggregates cultured with PC E8 lacking one protein at a time showed that (**i–k**) lack of bFGF, TGFβ1 or transferrin, omitted before conditioning, combined with PC-induced insulin loss elicited pronounced cell-disaggregation readily after 24 h of cultivation, (**m–o**) further progressing towards aggregate disruption on day 3, particularly when (**i,m**) insulin and bFGF were missing. In contrast, (**l**,**p**) omitting insulin ahead of PC reflected the respective (**d**,**h**) SC condition. (**q**) PI-based flow cytometry, performed for quantitative assessment of viability of day 3 hiPSC aggregates cultured in SC E8 lacking either bFGF, TGFβ1 or transferrin, showed dying PI-positive cells ranging from 4.5% to 6.3%, comparable to the 6.0% of the SC E8 control, while in the absence of insulin almost a 40% of dead cells was reached. Flow cytometry based on the pluripotency-associated markers (**r**) NANOG and (**s**) OCT4 performed on day 3 hiPSC aggregates revealed minor differences in marker expressions among hiPSC aggregates cultured with SC E8 and SC E8 lacking one protein at a time. (**t,u**) Insulin retrospectively supplemented to PC E8 medium allowed to support hiPSC aggregate cultivation. Scale bar = 100 µm.

To further correlate the physicochemical results with biological effects, hiPSC aggregates were cultured in SC E8 (control) and PC E8 lacking one protein at a time (omitted before initiation of conditioning, Fig. 4a–p). Interestingly, in SC E8 omission of either bFGF, TGFβ1 or transferrin had only minor effects on hiPSC aggregate morphology monitored for up to 3 days (Fig. 4a–c and e–g). In contrast, omitting insulin resulted in apparent shading of hiPSC aggregates readily after 24 h and progressive shrinkage thereafter (Fig. 4d and h). The morphological analysis was complemented by propidium iodide (PI)-based flow cytometry, revealing that the lack of either bFGF, TGFβ1, or transferrin induced 4.5% to 6.3% of PI-positive (*i.e*. dead) cells comparable to 6.0% observed in SC E8 controls (Fig. 4q). In contrast, in the absence of insulin, the percentage of dead cells reached 39.6% (Fig. 4q). This highlights the absolute necessity of insulin for hPSC integrity, at least for the applied culture conditions. On the other hand, omission of any other factor, including bFGF and TGFβ1 known to be essential for long-term maintenance of hPSCs^17, 38, 39^, was unremarkable in this short-term assay. This was further confirmed by the flow cytometry analysis of pluripotency-associated markers NANOG and OCT4, revealing minor differences in hiPSC aggregates cultured for 3 days in SC E8 versus SC E8 lacking one protein at a time (Fig. 4r and s), in accordance to literature^17, 40, 41^. However, combining the omission of specific media components before conditioning with peristaltic treatment induced much more drastic effects (Fig. 4i–p). Since peristaltic pump treatment depleted dissolved insulin from E8 (as demonstrated above by UPLC-MS investigation; Fig. 3o), the omission of bFGF, TGFβ1 or transferrin ahead of the conditioning process was now complemented with the PC-induced insulin loss. In consequence, presumptive lack of two factors provoked pronounced cell-disaggregation readily after 24 h of cultivation (Fig. 4i–k), further progressing towards aggregate disruption on day 3 (Fig. 4m–o). This was particularly evident when insulin and bFGF, both critical for hPSC survival^38^, were missing (Fig. 4i and m).

In contrast, omitting insulin ahead of peristaltic pump treatment essentially reflected the respective static conditioning (as shown in Fig. 4l and p versus Fig. 4d and h, respectively). This observation tallied with the idea that if insulin is not added to E8 beforehand any subsequent conditioning has a minor effect, if any, on media composition.

As additional control experiment, insulin was retrospectively supplemented to PC E8 medium, which therefore contained precipitated particles. Notably, the resulting medium regained ability to support hiPSC aggregate cultivation (Fig. 4t and u). This experiment underscored that the biological effects described herein were mainly induced by the depletion of insulin, whereas the presence of particle-like precipitates did not show obvious toxicity.

### Physical mechanisms occurring within and on the peristaltic pump-compressed tubing induced insulin precipitation

To investigate the mechanisms underlying insulin precipitation, E8 medium was tested at different flow rates within the peristaltic pump circuit, and by combining an alternative circulation set-up with different tubing formulations.

Flow rates of 5, 50 or 100 mL/min were tested for 12 h. Light microscopy images of PC E8 and consecutively cultured hiPSC aggregates showed slight differences depending on the applied flow rate, but the critical effect of peristaltic pumping was evident and comparable for all tested conditions (Supplementary Figure S3).

To independently investigate the medium-tubing interplay (by comparing two different tubing formulations, *i.e*. Platinum-cured silicone versus Tygon) and the role of mechanical stresses arising from tubing compression by the pump, a comparison between peristaltic (PC)- and chandler loop (CC)-based conditioning (see schematic in Fig. 1b) was performed applying the same flow rate. For both tested tubing formulations, no particles formed in CC E8 (Supplementary Figure S4a,b) and, on day 3, normal morphology and size distribution of cell aggregates equivalent to SC E8 controls were observed (Supplementary Figure S4e,f versus Fig. 2c). In contrast, precipitated particles were detected within the PC E8 for both tubing types (Supplementary Figure S4c,d) and detrimental biological effects on hiPSC aggregates were found at day 3 (exemplary depicted in Supplementary Figure S4g,h). Scanning electron microscope (SEM) images of the tubing lumen (Supplementary Figure 5) showed the effect of the peristaltic pump on the tubing. Tubing sections collected from regions outside the pump head were smooth and homogeneous at their internal surface (Supplementary Figure 5a–c). In contrast, amorphous agglomerates attached to the tubing lumen were detected in samples from the pump head region (Supplementary Figure 5d–f) and identified as silicone debris (Supplementary Figure S6), showing that continuous cyclic compression caused local tubing abrasion. These results suggested that insulin precipitation was promoted by the combination of hydrodynamic and mechanical forces acting within and on the tubing segment located in the peristaltic pump head, and the interfacial interaction of the media with the hydrophobic inner surface of the tubing, characterized by increased roughness due to wear.

### Insulin depletion and hPSC viability were rescued by HSA supplementation

**Figure 5:**
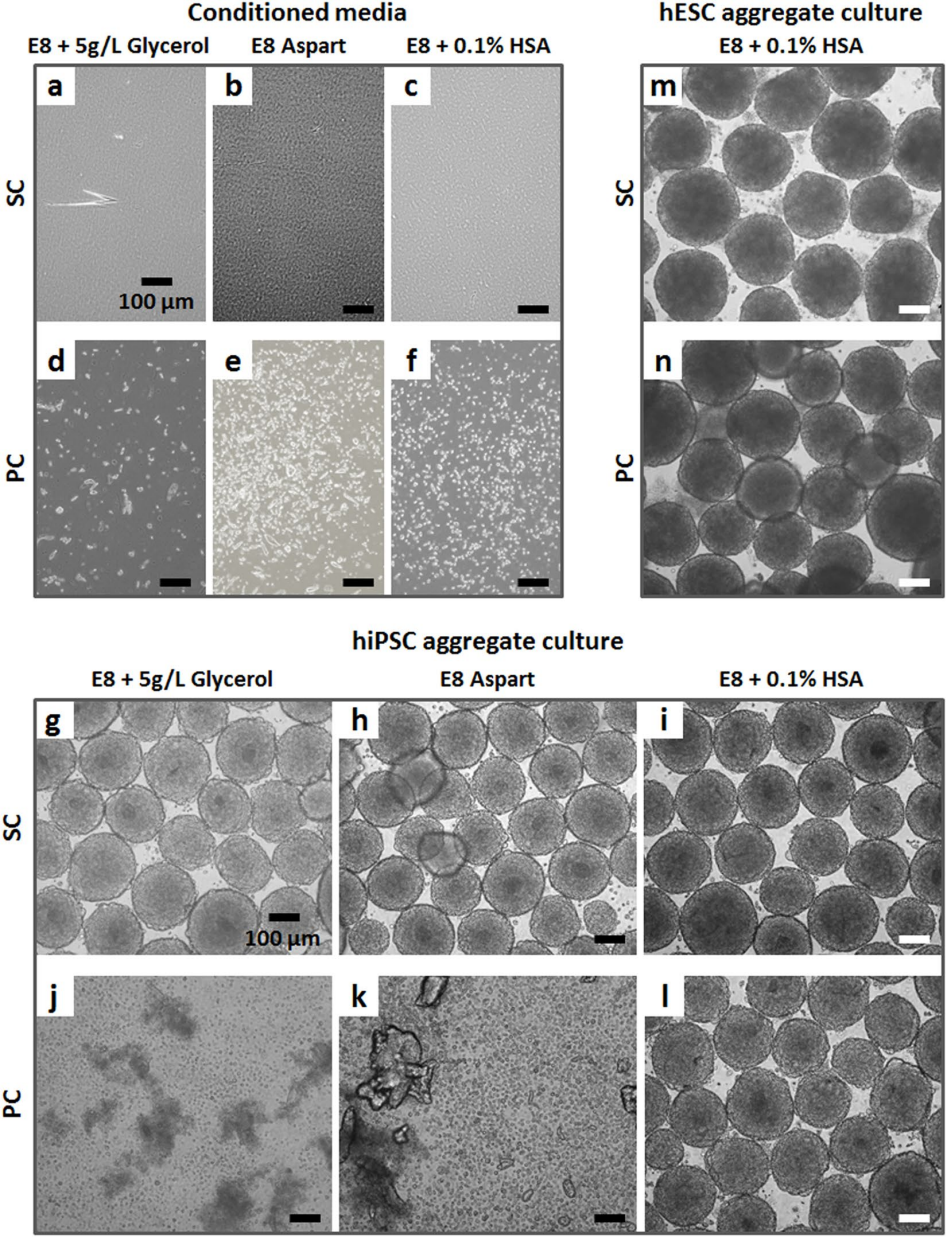
hPSC aggregates cultured in suspension with SC E8 and PC E8 supplemented with glycerol, Aspart or HSA. Light microscopy images of (**a**) SC E8 + 5 g/L Glycerol, (**b**) SC E8 Aspart, and (**c**) SC E8 + 0.1% HSA showed transparent media that (**g**–**i**) did not affect the hiPSC aggregate culture, similarly to (**m**) hESC aggregates cultured with SC E8 + 0.1% HSA. In contrast, (**d**) PC E8 + 5 g/L Glycerol, (**e**) PC E8 Aspart, and (**f**) PC E8 + 0.1% HSA did not prevent particle precipitation, and neither (**j**) glycerol nor (**k**) Aspart were able to maintain hiPSC culture. However, the addition of HSA efficiently supported (**l**) hiPSC and (**n**) hESC propagation in suspension. Scale bar = 100 µm.

Inspired by the pharmacological strategies based on excipients targeted to maximize stability of dissolved proteins, the following approaches were tested (Fig. 5): (1) addition of glycerol^42, 43^; (2) replacement of insulin commonly used for culture media formulation with the analogue insulin Aspart^44, 45^; (3) supplementation of human serum albumin (HSA)^33, 46, 47^.

Reflecting published studies^43, 48^, glycerol was added at 1, 5, or 10 g/L to E8 before conditioning; data shown for 5 g/L glycerol are representative for this assay. The resulting SC medium (E8 + 5 g/L Glycerol) appeared transparent (Fig. 5a) and glycerol addition did not affect the hiPSC aggregate culture (Fig. 5g). However, glycerol at any tested concentration did neither prevent PC-induced insulin precipitation (Fig. 5d), nor enabled hiPSC aggregate culture (Fig. 5j).

Next, the engineered insulin Aspart, approved for therapeutic use, was used for conventional insulin replacement (E8 Aspart). SC E8 Aspart (Fig. 5b) fully supported hiPSC aggregate culture (Fig. 5h). However, after peristaltic pump conditioning typical microscopic particles were detected (Fig. 5e) and the medium disrupted hiPSC aggregates cultivation (Fig. 5k).

Finally, HSA at 0.01%, 0.1%, or 1% was added to E8 before conditioning. The SC E8 + 0.1% HSA was transparent (Fig. 5c) and suitable for hiPSC (Fig. 5i) and hESC (Fig. 5m) aggregate culture. Surprisingly, although particle formation was detectable in the peristaltic conditioned medium (Fig. 5f), PC E8 + 0.1% HSA efficiently supported hiPSC (Fig. 5l) and hESC (Fig. 5n) propagation in suspension. A similar outcome but at lower efficacy was observed for hiPSC aggregates cultured with E8 + 0.01% HSA (Supplementary Figure S7e,g), while at higher concentration of 1% HSA detrimental effects on hiPSC aggregates for both static and peristaltic conditioning were observed (Supplementary Figure S7f,h).

**Figure 6:**
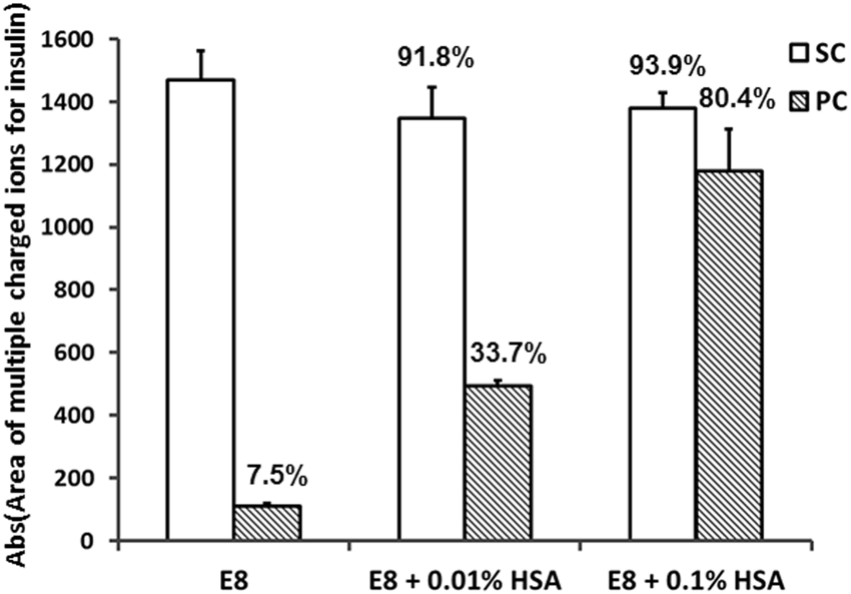
UPLC-MS analysis of SC E8 and PC E8 supplemented with HSA. The UPLC-MS analysis performed on samples of SC and PC E8, E8 + 0.01% HSA and E8 + 0.1% HSA revealed maintenance of high dissolved insulin content in PC E8 + 0.1% HSA (around 80%) compared to SC E8 controls and in contrast to PC E8 (7.5%), while dissolved insulin was slightly preserved in PC E8 + 0.01% HSA (>33% of insulin remaining) (n = 2).

Analysis by UPLC-MS (Fig. 6) revealed maintenance of high dissolved insulin content in PC E8 + 0.1% HSA (around 80%) compared to SC E8 and in contrast to the PC E8 (7.5%), while dissolved insulin was slightly preserved in PC E8 + 0.01% HSA (>33% of insulin remaining). Together, this supported the view that (1) insulin availability is the key factor for hiPSC survival in the adopted assay, and (2) HSA is beneficial for insulin preservation and stabilization.

**Figure 7:**
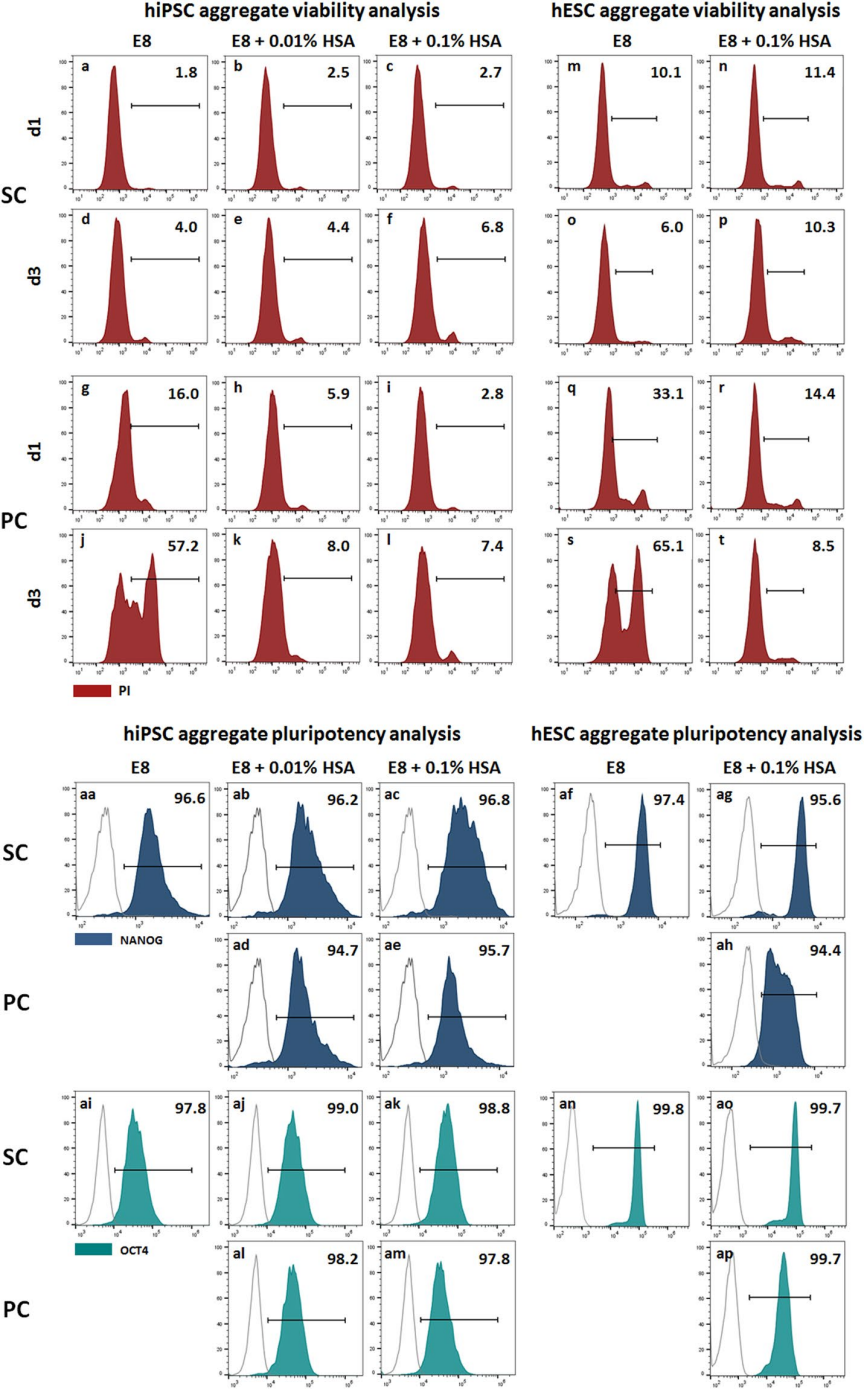
Viability and pluripotency of hPSC aggregates cultured in suspension with SC E8 and PC E8 supplemented with HSA. PI-based flow cytometry was performed on day 1 and day 3 for quantitative assessment of viability of hiPSC aggregates cultured with (**a–f**) SC and (**g–l**) PC E8, E8 + 0.01% HSA and E8 + 0.1% HSA, and of hESC aggregates cultured with (**m–p**) SC and (**q–t**) PC E8 and E8 + 0.1% HSA. A dead cell content of (**g**) 16% for hiPSC aggregates and (**q**) ~33% for hESC aggregates cultured in PC E8 was measured at day 1, reaching (**j**) ~60% and (**s**) ~65% on day 3, respectively, which is a drastic loss of viability compared to (**a**,**d**, and **m**,**o**, respectively) SC E8 controls. The presence of HSA substantially limited the percentage of dying PI-positive cells for (**h**,**i**,**k**, and **l**) hiPSC aggregates and for (**r**,**t**) hESC aggregates, in accordance with the qualitative assessment by light microscopy. Pluripotency assessment of day 3 hPSC aggregates revealed that more than 90% of cells expressed pluripotency-associated transcription factors NANOG for (**aa–ae**) hiPSCs and for (**af–ah**) hESCs and OCT4 for (**ai–am**) hiPSCs and for (**an–ap**) hESCs, confirming that HSA did not affect maintenance of pluripotency.

Quantitative assessment of hPSC viability by PI-based flow cytometry revealed, on day 1, a dead cell content of 16% for hiPSC (Fig. 7g) and ~33% for hESC (Fig. 7q) aggregates cultured in PC E8, while for SC E8 controls values of ~2% for hiPSCs (Fig. 7a) and ~10% for hESCs (Fig. 7m) were found. On day 3, ~57% PI-positive cells for hiPSC (Fig. 7j) and ~65% for hESC (Fig. 7s) aggregates were discovered, representing a drastic loss of viability compared to 4–6% for respective hiPSC (Fig. 7d) and hESC (Fig. 7o) controls cultured in SC E8. Moreover, this assay confirmed that the presence of HSA substantially limited the percentage of PI-positive cells for both hPSC aggregates, *e.g*. on day 3 cell death was limited to ~7–8% for hiPSCs (Fig. 7k and l) and ~9% for hESCs (Fig. 7t), in accordance with the qualitative assessment of aggregates by light microscopy (Supplementary Figure S7g and Fig. 5l and n).

Finally, pluripotency assessment of day 3 hPSC aggregates revealed that more than 90% of cells expressed NANOG (Fig. 7aa–ae for hiPSCs and Fig. 7af–ah for hESCs) and OCT4 (Fig. 7ai–am for hiPSCs and Fig. 7an–ap for hESCs) with negligible differences among SC E8 with/without HSA and PC E8 + HSA (Fig. 7). This further suggested that HSA did not affect maintenance of pluripotency.

### Commercial high protein media supported hiPSC viability upon peristaltic pump conditioning

To ensure general validity of the presented results (beyond self-made production of E8), three commercial media for hPSC maintenance were tested.

**Figure 8:**
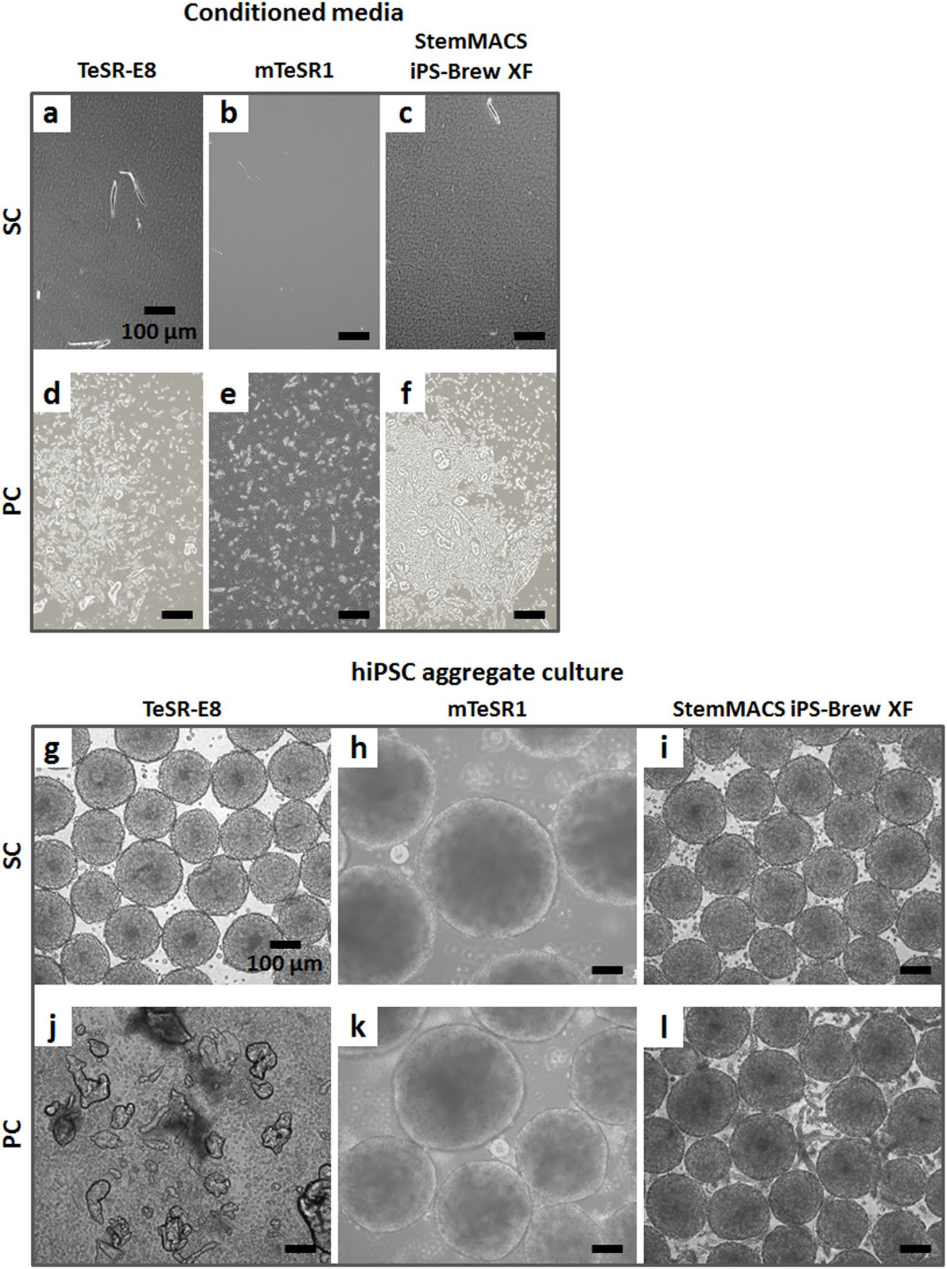
hiPSC aggregates cultured in suspension with SC and PC commercial media. Light microscopy images of (**a**) SC TeSR-E8, (**b**) SC mTeSR1, and (**c**) SC StemMACS iPS-Brew XF showed transparent media, while (**d**) PC TeSR-E8, (**e**) PC mTeSR1, and (**f**) PC StemMACS iPS-Brew XF were all characterized by particle precipitation. Concerning the hiPSC aggregate culture, (**g**) low protein TeSR-E8 supported the culture when static conditioned, but (**j**) it caused hiPSC disruption when peristaltic pump conditioned. Contrarily, static and peristaltic pump conditioning of high protein media supported hiPSC aggregate culture, even if with (**h**,**k**) hiPSC aggregates more heterogeneous in size in case of mTeSR1, and (**i**,**l**) elevated amount of particles in case of StemMACS iPS-Brew XF. Scale bar = 100 µm.

TeSR-E8^49^, a low protein medium based on E8, supported hiPSC aggregate culture when statically conditioned (Fig. 8g), as expected, but peristaltic pump conditioning induced particle precipitation (Fig. 8d) and loss of aggregate integrity (Fig. 8j).

Peristaltic conditioning of high protein media mTeSR1 (BSA supplemented^50^) and StemMACS iPS-Brew XF induced noticeable micro-particle precipitation as well (Fig. 8e and f, respectively), but still supported hiPSC aggregate culture (Fig. 8k and l, respectively). However, hiPSC aggregates cultured in SC and PC mTeSR1 were more heterogeneous in size compared to SC E8 control (Fig. 8h and k versus Fig. 2c, and Supplementary Figure S8), while in PC StemMACS iPS-Brew XF a considerably elevated amount of precipitated particles became obvious (Fig. 8l) compared to PC E8 + 0.1% HSA controls (Fig. 5l).

## Discussion

In this study, specific hPSC media broadly adopted for hPSC suspension culture^3, 4, 17, 21, 49^ were tested for continuous peristaltic pump-based circulation. Peristaltic pumping is a key technology extensively applied for perfusion and hydraulically-driven bioreactors since it enables closed systems with no risk of contamination by air or the direct contact with pump components^29–32^. The ultimate aim of this study was to assess compliance of hPSC media with a recently published peristaltic pump-based, impeller-free suspension bioreactor^27^ in the perspective of future hPSC culture.

The investigations revealed that hPSC media are sensitive to the adopted operating conditions. In particular, when the low protein media E8 and TeSR-E8 were circulated in a peristaltic pump circuit, precipitation of insoluble particles (Figs 3c,d and 8d) and a marked insulin depletion were observed (Fig. 3o). The drastic reduction of soluble insulin induced a severe viability loss in day 3 hPSC aggregates cultured in suspension serving as a relevant reference assay (Figs 2d–f,j–l, 7g,j,q,s and 8j). Notably, the addition or the presence of bovine or human serum albumin in the hPSC media (*i.e*. E8 + HSA, mTeSR1 and StemMACS iPS-Brew XF) did not prevent the precipitation of particles upon peristaltic pumping (Figs 5f, 8e and f), but rescued soluble insulin (see Fig. 6 for E8 + HSA), ultimately aiding hPSC culture (Figs 5l,n, 7l,t and 8k and l).

Due to its extensive therapeutic use for diabetic patients, insulin is one of the most widely studied molecules in biochemistry. Its tendency to undergo structural transformation resulting in the formation of insoluble particles when exposed to physicochemical stresses, also called insulin self-association or fibrillation, is well-known in pharmaceutical research and industry^33, 51^. Insulin precipitation can lead to a drastic reduction in its biological and therapeutic potency, representing a critical issue during pharmaceutical processing, transport, storage, and clinical administration through delivery devices^52–55^.

Even though the exact mechanism of insulin fibrillation is still not fully understood, it is clear that it occurs through a series of sequential physical and/or chemical degradation pathways^35, 54^, leading to the formation of biologically inactive particles^33, 51^. In detail, the initial step in aggregation is probably the formation of monomeric molecules characterized by exposed hydrophobic faces, normally buried in the 3D structure^35, 51, 52^. The resulting non-native insulin molecules are prone to mutual interaction, with a high probability to form amorphous particles or organized fibrils that ultimately compromise the safety and efficacy of the protein^33, 35, 51^.

Different physicochemical factors (*e.g*. temperature, motion, mechanical stress, interaction with hydrophobic surfaces, metal ions, pH, *etc*.) have been identified to promote insulin precipitation^33, 51, 53, 56^. Particularly, in delivery devices (*e.g*. artificial pancreas or portable/implantable infusion pumps) or during production processes (*e.g*. ultrafiltration, diafiltration, *etc*.) the combination of fluid mechanics forces, interaction with hydrophobic surfaces, and heat can promote insulin fibrillation^54, 55^.

Though insulin fibrillation has been extensively investigated in pharmacological research, to the best of our knowledge, this is the first study providing evidence that continuous peristaltic pump-based circulation of low protein hPSC expansion media provokes insulin precipitation and depletion and consequently induces severe hPSC culture failure.

In recent years, increasing effort has been dedicated to identify the optimal hPSC culture conditions for defining robust chemically defined systems, and several media based on signal proteins and growth factors (*e.g*. insulin, transferrin, albumin, bFGF, TGFβ1, Wnt, IGF), lipids, vitamins and additives were proposed^17, 18, 50, 57–61^. On the one hand, it is known that the self-renewal of hESCs requires insulin-like growth factor-1 (IGF-1) receptor signaling^59^ and that IGF-II has a central regulatory role in mammalian pre-implantation and embryonic development^62^ with high sensitivity of human neural stem cells and neurons to insulin concentration^63^. On the other hand, to the best of our knowledge, no in-depth investigation on the specific role and concentration requirements of insulin for hPSC viability and proliferation is yet available, requesting further attention. This may include the parallel analysis of other media components, particularly regarding the interplay of the insulin-to-glucose ratio and its impact on the hPSC metabolism. Recently an adaptive, process-dependent flexibility of the hPSC metabolism at the pluripotent state was reported^21^, and metabolic changes (*i.e*. glycolysis vs. oxidative phosphorylation) during hPSC differentiation and somatic cell reprograming into hiPSC are well established^64, 65^. The known inhibitory role of insulin, for example, during cardiac mesoderm induction of hPSCs^66, 67^ and the effects on viability at the pluripotent state shown in this study strongly mandate process-dependent adaptation and monitoring of the insulin concentration in the future.

In accordance with previous studies on insulin aggregation mechanisms in agitated aqueous solutions^54, 68–70^, findings in this study demonstrate that the underlying mechanisms inducing insulin fibrillation include: (1) hydrodynamic and mechanical forces acting within and on the tubing located in the peristaltic pump head, in combination with (2) the interaction of the media with the hydrophobic surface of the tubing lumen, possibly catalyzed by (3) its increased roughness, as a consequence of tubing wear caused by the cyclic compression. Technically, the medium flows through flexible tubing which, in the peristaltic pump region, is fitted into a circular casing where rollers, connected to a rotating rotor, cyclically compress it. The portion under compression is occluded, forcing the fluid to cyclically undergo high pressures and shear stresses and to move ahead through the tubing^28^. Moreover, local heating might result from the locally-induced turbulent kinetic energy dissipation in the medium. These complex fluid mechanics phenomena, in combination with a constant interaction of the medium with the hydrophobic surface of the tubing, could stress insulin. Subsequently, self-association of insulin into amorphous insoluble particles is fostered depleting soluble insulin.

Definitive strategies for dissolved insulin stabilization have not been established^35^. However, several stabilizing additives have been described including: ionic and non-ionic surfactants^53, 54^, glycerol^42, 43^, albumin^47, 71^, α-crystallin^35^. Furthermore, alternative insulin formulations, including artificial insulin analogues, have been proposed^43, 45, 48^.

In this study, three different strategies to avoid dissolved insulin depletion from E8 were followed. Firstly, the addition of glycerol: the compound did not affect hiPSC aggregate culture (Fig. 5g) but could not overcome insulin precipitation neither rescue hPSC viability in case of peristaltic pump conditioning (Fig. 5d and j). Secondly, an artificial insulin analogue, Aspart, was tested. Developed for continuous subcutaneous insulin infusion pumps^44^, Aspart is characterized by reduced tendency of molecular self-association compared to human insulin^45^. While E8 supplemented with Aspart fully supported hiPSC aggregate culture in controls (Fig. 5h), its precipitation induced by peristaltic pumping was not avoided (Fig. 5e,k).

Lastly, albumin supplementation, which has been extensively used as stabilizing excipient in several therapeutic protein formulations^46, 56^, was tested. Although micro-particles were detectable in PC E8 + HSA (Fig. 5f, Suppl. Fig. S7c,d) and HSA itself may precipitate as well^35^, 0.1% HSA did preserve insulin under peristaltic pump conditioning (Fig. 6) and efficiently supported hPSC propagation (Figs 5l and 7l for hiPSCs, Figs 5n and 7t for hESCs) without affecting pluripotency maintenance (Fig. 7ae and am for hiPSCs, Fig. 7ah and ap for hESCs). Mechanistically, HSA may (1) counteract the adsorption of insulin to the tubing surface^33–35^, and/or (2) stabilize insulin by acting as a molecular chaperone preventing the misfolding and/or aggregation into precipitated particles^47, 71^.

In this study non-recombinant HSA was adopted. However, in order to develop clinically-compliant culture conditions for future applications, recombinant human serum albumin (rHSA) should be considered to guarantee high level of purity and to avoid batch variability and possible contamination by viruses or prions^46, 72^.

Pump-based circuits are broadly applied in biotechnology. This includes their use in automated monitoring devices^73^ as well as for specific bioprocessing methods and cell culture bioreactors^9, 27, 74–76^. This study demonstrates how the application of such established technology may result in unexpected, previously overlooked issues, when applied in a closely related field. The presented findings put the spot on the important aspects of hPSC cultivation in low-protein media, which is generally favored for the production of Advanced Therapy Medicinal Products (ATMPs), such as hPSC-derived progenies.

In the perspective of developing chemically defined culture media and establishing novel strategies for automated propagation of hPSCs, the inherent physical instability of insulin may become a substantial hurdle. In more general terms, the study highlights the importance of closely monitoring culture media components in frame of the applied culture strategy. While this is already applied in process development of conventional mammalian mass cell culture^77^, this topic has been hardly attended in the field of hPSC cultivation and differentiation.

## Methods

### Materials

hPSC culture media: self-made chemically defined E8^17^, (Supplementary Table S9); TeSR-E8 (STEMCELL Technologies, Vancouver, Canada); mTeSR1 (STEMCELL Technologies, Vancouver, Canada); StemMACS iPS-Brew XF (Miltenyi Biotec GmbH, Bergisch Gladbach, Germany). Further culture media: Gibco DMEM/F12 (Thermo Fisher Scientific Inc., MA, USA); self-made E8 lacking one protein at a time (*i.e*. E8 minus -bFGF, -TGFβ1, -transferrin, -insulin); self-made E8 supplemented with 1, 5, or 10 g/L of glycerol (Sigma-Aldrich, Munich, Germany); E8 containing 20 mg/mL insulin analogue Aspart (Novorapid, Novo Nordisk A/S, Bagsvaerd, Denmark) for replacement of conventional human insulin; E8 supplemented with 0.01%, 0.1%, or 1% of HSA (Biological Industries, Beit-Haemek, Israel). Cell culture: Geltrex (Thermo Fisher Scientific Inc., MA, USA); self-made Rho-associated coiled-coil kinase inhibitor Y27632 (RI)^78^. Flow cytometry: propidium iodide (PI) (BD Biosciences, Franklin Lakes, NJ, USA), NANOG (Cell Signaling Technology, Danvers, MA, USA), OCT4 (Santa Cruz Biotechnology, Santa Cruz, CA, USA), Cy-3 conjugated Donkey anti-Rabbit and Cy-5 conjugated Donkey anti-Mouse (Jackson ImmunoResearch Inc., West Grove, PA, USA). Tubing: Biopharm Plus Platinum-cured Silicone (Masterflex, Cole-Parmer, IL, USA); Tygon E-LFL (Masterflex, Cole-Parmer, IL, USA).

### Culture media conditioning

The hPSC media were conditioned overnight in the incubator (37 °C, 5% CO_2_) under (Fig. 1b): (1) static conditioning in 50 mL tube (SC); (2) continuous circulation within a closed-loop peristaltic pump-based circuit (PC). The PC circuit was composed of a medium reservoir, oxygen-permeable platinum-cured silicone tubing with quick-disconnect couplings, and a peristaltic pump (Masterflex L/S Tubing Pump, Cole-Parmer, IL, USA), for a total working volume of approximately 50 mL. Within the PC circuit, the culture media were conditioned imposing flow rates of 5, 50 and 100 ml/min. In addition, culture medium E8 was tested using two different tubing formulations (Biopharm Plus Platinum-cured Silicone and Tygon E-LFL) and conditioned under continuous circulation within a chandler loop system (CC, Fig. 1b). In the CC set-up, the continuous circulation of the medium was obtained with a closed loop tubing imposing a rotational velocity set to maintain the same PC flow rate.

### hPSC aggregate culture

Biological experiments were performed using the hiPSC line hHSC_1285_T-iPS2^79^, obtained from our laboratory upon request and derived from peripheral blood donated by healthy volunteers following informed consent, and the hESC line HES3 NKX2-5eGFP/w^80^, available from Dr. Elliott (Murdoch Childrens Research Institute). Ahead of process inoculation, hPSCs were expanded in feeder-free monolayer culture on Geltrex-coated flasks in E8 for at least 4 passages as described in detail elsewhere^26^. Pre-cultures for the tests did not exceed 8 passages in feeder-free monolayer culture.

In order to obtain hPSC aggregates, single cells were cultured in 3 mL E8 supplemented with the RI (10 µM) small molecule within 6-well suspension culture plates (1x10^6^ cells/well, d-1) positioned on an orbital shaker (70 revolution per minute = rpm) for 24 h inside the incubator^5^. hPSC aggregates were harvested (d0) and cultured with conditioned media in 6-well suspension culture plates positioned on an orbital shaker (70 rpm) for further 72 h (Fig. 1a). No media changes were performed after d0 and for the 3 days of culture. All tests were performed in three independent runs. hPSCs were used in accordance with the Hannover Medical School Ethics Committee and are covered by appropriate consent that permits their use in the proposed research. All procedures were performed in accordance with the applicable ethical and legal regulations.

### Light microscopy

Light microscopy images were recorded (1) right after the conditioning for media inspection, and (2) daily for monitoring the hPSC aggregates (Axio Vert.A1 microscope and Axiovision 8.4 software, Zeiss, Thornwood, NY).

### Flow cytometry

Flow cytometry analyses for characterizing cell death (PI) at day 1 and day 3, and cell pluripotency (NANOG, OCT4) at day 3 have been performed. hPSC aggregates were harvested, washed with PBS and dissociated into single cells with Accutase. Following cell count 2x10^5^ single cells were stained with PI according to manufacturer’s instructions. NANOG and OCT4 staining were performed as follow: 2x10^5^ single cells were fixed with 90% methanol for 15 min on ice, then washed thoroughly and primary antibody added for one hour at room temperature according to manufacturer’s dilution. Following a washing step, secondary antibodies were added (Cy-3 conjugated Donkey anti-Rabbit for NANOG, Cy-5 conjugated Donkey anti-Mouse for OCT4) according to manufacturer’s dilution and incubated at room temperature for one hour in the dark. Buffer used for antibody addition was composed of PBS with 0.5% BSA, 3 mM EDTA and 0.1% Triton X-100. Samples suspended in PBS with 0.5% BSA and 3 mM EDTA were then assessed with the BD Accuri C6 Flow Cytometer, gates were set according to respective isotype controls.

### UPLC-MS analysis

UPLC-MS analyses were performed on the conditioned media to provide a semi-quantitative measure of the remaining concentration of insulin. Each sample was separated by UPLC (Waters Acquity UPLC; column Waters Acquity HSS T3 1.8 µm, 2.1 × 100 mm; solvent A = water, 0.1% formic acid; solvent B = acetonitil, 0.1% formic acid; solvent gradient: 0 min (98% A), 2 min (98% A), 9 min (50% A), 12 min (0% A), 13 min (0% A), 13.10 min (98% A), run time 15 min; injection volume 5 mL, flow rate = 400 µL/min) and analysed by ESI-MS (Waters Q-Tof premier, positive mode, capillary voltage = 3 kV). The insulin content was determined by recording and integrating the intensity of the most intensive multiple charged positive ions for insulin (4,5,6-time charged ions 968.8, 1162.4, and 1452.1, respectively; n = 2).

### Data availability

The datasets generated and/or analyzed during the current study are available from the corresponding author on reasonable request.

See Supplementary Information for details about scanning electron microscope analysis of the tubing lumen and characterizations of the PC E8 precipitated particles and of the agglomerates attached to the tubing lumen.

## Electronic supplementary material

Supplementary Information
