# Supplementary material for: Sensitivity of human pluripotent stem cells to insulin precipitation induced by peristaltic pump-based medium circulation: considerations on process development
Source: Sci Rep. 2017 Jun 21;7:3950. doi: 10.1038/s41598-017-04158-x (PMC5479836; doi:10.1038/s41598-017-04158-x)
Supplement: Supplementary file 1 — Supplementary Information [file 41598_2017_4158_MOESM1_ESM.pdf]

Sensitivity of human pluripotent stem cells to insulin precipitation induced by peristaltic pump-based medium circulation: considerations on process development

Diana Massai[1,2], Emiliano Bolesani[1,2], Diana Robles Diaz[1,2], Christina Kropp[1,2], Henning Kempf[1,2], Caroline Halloin[1,2], Ulrich Martin[1,2], Tudor Braniste[1,3], Giuseppe Isu[4,5], Vanessa Harms[6], Umberto Morbiducci[4], Gerald Dräger[2,6], Robert Zweigerdt[1,2*]

1 Leibniz Research Laboratories for Biotechnology and Artificial Organs (LEBAO), Department of Cardiac, Thoracic-, Transplantation and Vascular Surgery, Hannover Medical School, Carl-Neuberg-Str. 1, 30625 Hannover, Germany

2 REBIRTH-Cluster of Excellence, Hannover Medical School, Carl-Neuberg-Str. 1, 30625 Hannover, Germany

3 National Center for Materials Study and Testing, Technical University of Moldova, bv. Stefan cel Mare 168, Chisinau MD-2004, Republic of Moldova

4 Department of Mechanical and Aerospace Engineering, Politecnico di Torino, Corso Duca degli Abruzzi 24, 10129 Torino, Italy

5 Department of Biomedicine, University of Basel and Department of Surgery, University Hospital of Basel, 4031, Basel, Switzerland

6 Institute of Organic Chemistry, Leibniz University Hannover, Schneiderberg 1B, 30167 Hannover, Germany

* Corresponding author: Robert Zweigerdt, Leibniz Research Laboratories for Biotechnology and Artificial Organs (LEBAO), Hannover Medical School, Carl-Neuberg-Str. 1, 30625 Hannover, Germany. Phone: +49 511 532 5023, Fax: +49 511 532 8819, Email: zweigerdt.robert@mh-hannover.de

Characterization of the PC E8 precipitated particles

To characterize the particles precipitated in the peristaltic conditioned E8 medium a published method for the solubilization of insulin amyloid fibrils [Nilson&Dobson, 2003] has been applied. In detail, precipitated particles harvested upon centrifugation from a falcon containing 40 mL of PC E8 (30 min, 8500 rpm, 4 °C) were incubated with 7N ammonia for 30 min at room temperature. The supernatant was analyzed by LC-MS (Waters QTof premier ESI-MS coupled with Waters Acquity UPLC; Waters Acquity UPLC BEH Phenyl column with 1.7 µm particles and 2.1 mm * 100 mm column dimensions; linear gradient of water with 0.1% formic acid (A) and acetonitril with 0.1% formic acid (B) (0 min = 10% B; 6 min = 100% B; 6.5 min = 100% B, 6.6 min = 10% B, 8 min = 10% B; runtime = 8 min; flow rate = 0.4 mL/min)). Insulin shows a retention time of 2.25-2.50 min and mainly five times charged (m/z = 1161.9) and four times charged (m/z = 1452.5) ions. The ESI spectra for this time range were summarized and deconvoluted using Maxent (part of Masslynx software package, Waters). The LC-MS analysis of the harvested precipitated particles, partially solubilized in 1 mL 7N ammonia solution, proved the presence of insulin (Supplementary Figure S1). The particles were not washed before ammonia treatment and still contained 70 µL of residual medium. Therefor 70 µL of the clear supernatant were also treated with 7N ammonia and this sample contained only traces of insulin (Supplementary Figure S2).

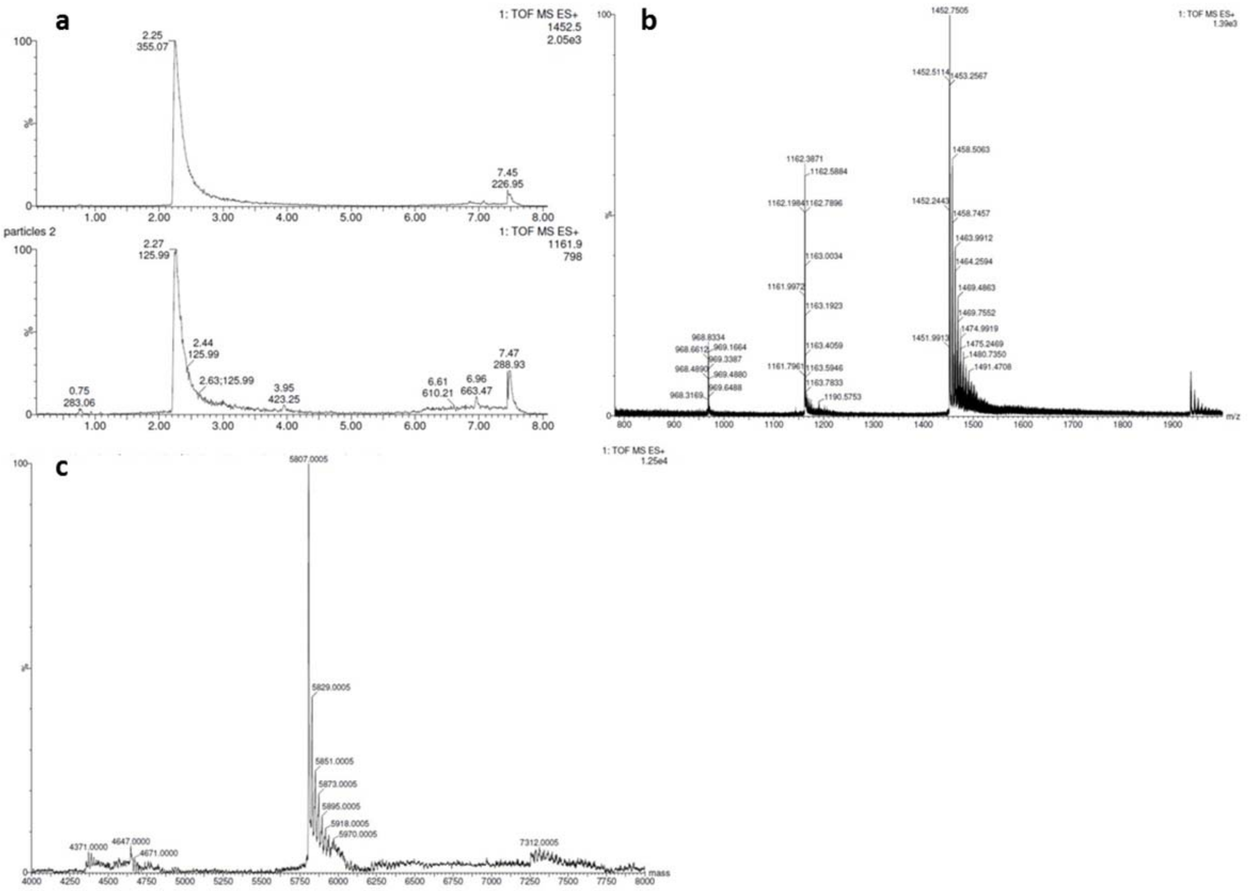

Supplementary Figure S1. Characterization of the PC E8 precipitated particles. (**a**) Selected ion chromatogram for m/z = 1161.9 and 1452.5. (**b**) Summarized ESI-spectra 2.25-2.5 min. (**c**) Deconvoluted ESI spectra 2.25-2.5 min.

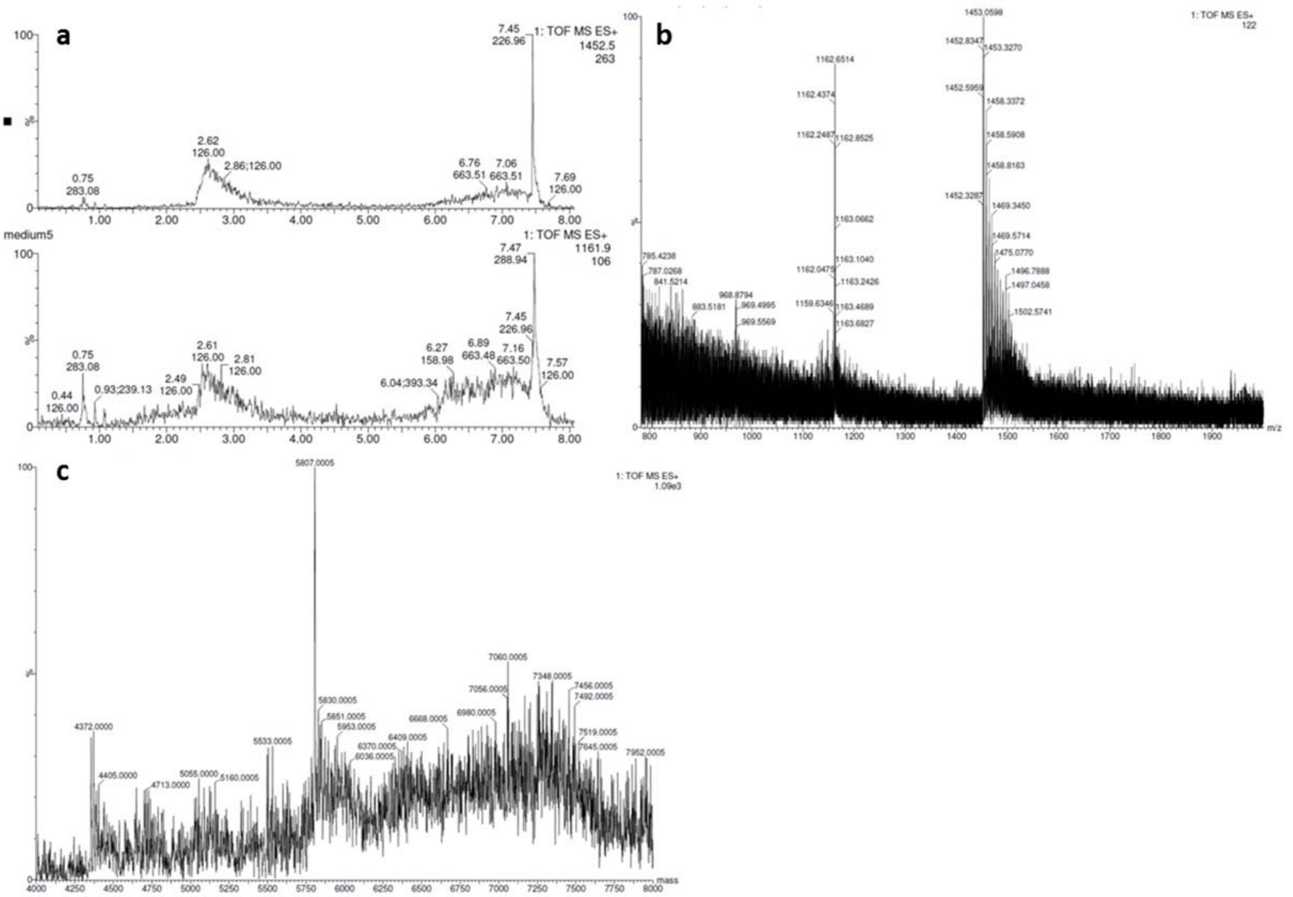

Supplementary Figure S2. Characterization of the supernatant. (**a**) Selected ion chromatogram for m/z = 1161.9 and 1452.5. (**b**) Summarized ESI-spectra 2.25-2.5 min. (**c**) Deconvoluted ESI spectra 2.25-2.5 min.

References

Nilsson, M. R., & Dobson, C. M. Chemical modification of insulin in amyloid fibrils. *Protein Science*, **12**, 11, 2637–2641 (2003).

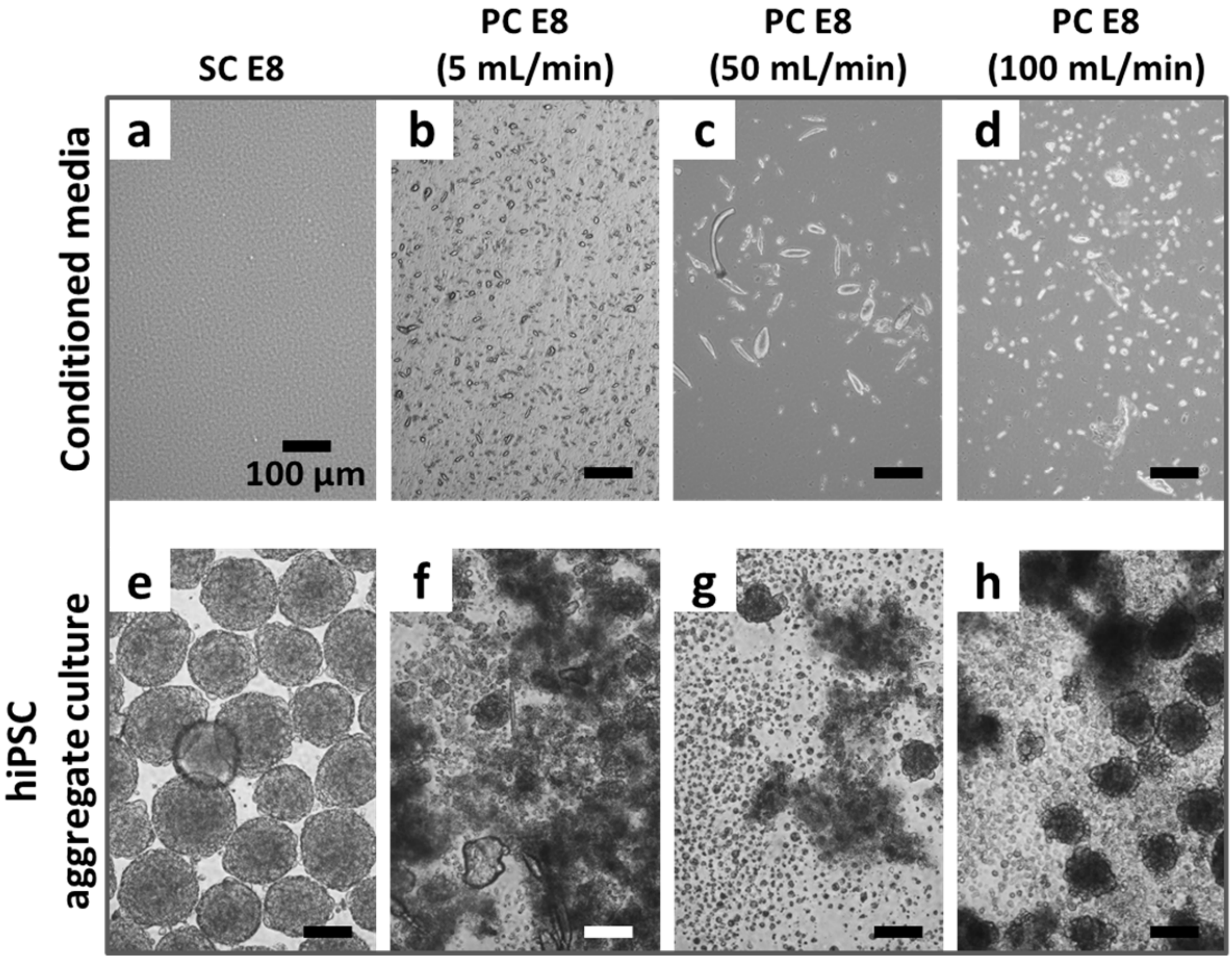

Supplementary Figure S3. Effect of different PC E8 flow rates. Light microscopy images of (**a**) SC E8 and PC E8 imposing flow rates of (**b**) 5, (**c**) 50 or (**d**) 100 mL/min. hiPSC aggregates cultured in (**e**) SC E8, and in PC E8 imposing flow rates of (**f**) 5, (**g**) 50 or (**h**) 100 mL/min. Microscopic precipitates formed in all the PC E8 media with comparable biological effect of suspended single cells and substantial disaggregation of cultured hiPSC aggregates. Scale bar = 100 µm.

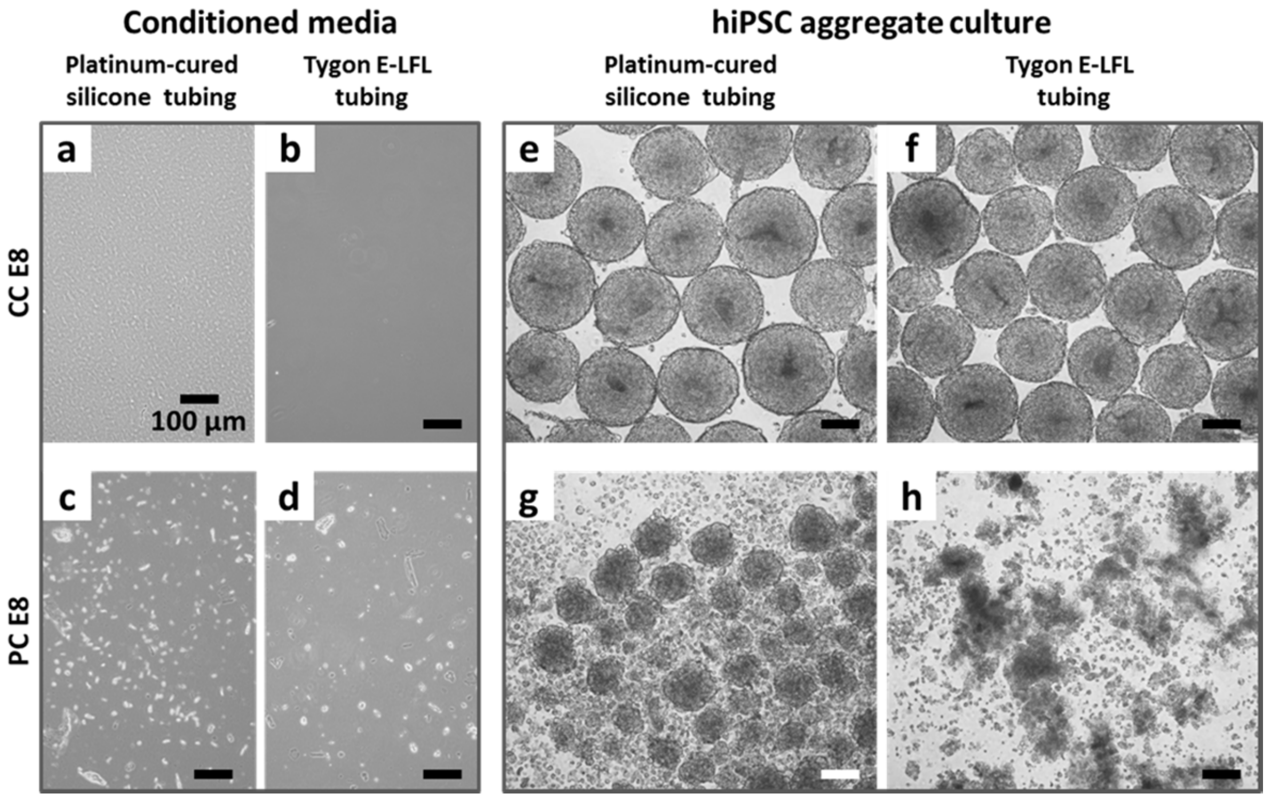

Supplementary Figure S4. Effect of CC and PC E8, using different tubing formulations. Light microscopy images of CC E8 circulated within (**a**) Platinum-cured silicone tubing and (**b**) Tygon tubing. (**e, f**) As regards CC E8, no particles formation was observed for both tubing formulations, with normal morphology and size distribution of cell aggregates at day 3 equivalent to SC E8 controls. In contrast, precipitated particles were detected within the PC E8 for both (**c**) Platinum-cured silicone tubing and (**d**) Tygon tubing. (**g, h**) Detrimental effects on hiPSC aggregates were observed as exemplified in respective pictures. Scale bar = 100 µm.

Scanning electron microscopy of the tubing lumen

Scanning electron microscope investigations (Philips-505, Acceleration Voltage 10 kV) of two sections of the tubing lumen, coming respectively from the reservoir region (far from the pump) and from the pump head region, were recorded. Approximately 10 nm of elementary gold was sputtered on the surface of the tubing samples using a Polaron SEM Coating System in order to avoid charging effects during scanning.

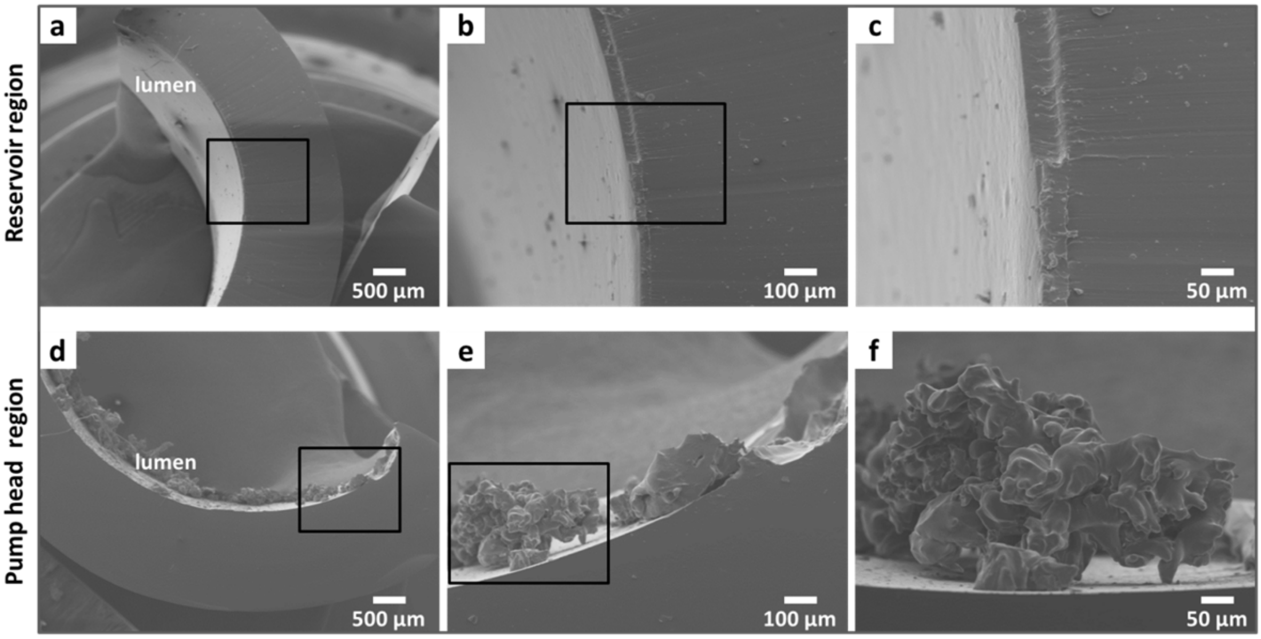

Supplementary Figure S5. Scanning electron microscopy of the tubing lumen. (**a**) The SEM image of the tubing lumen from region outside the pump head showed a smooth and homogeneous internal surface (scale bar = 500 µm). (**b**) Higher magnification (rectangle in a) of the surface of the lumen (scale bar = 100 µm). (**c**) Higher magnification (rectangle in b) of the surface of the lumen (scale bar = 50 µm). (**d**) The SEM image of the tubing lumen from the peristaltic pump head region showed amorphous agglomerates attached to the tubing lumen. (**e**) Higher magnification (rectangle in d) of the surface of the lumen (scale bar = 100 µm). (**f**) Higher magnification (rectangle in e) of the surface of the lumen (scale bar = 50 µm).

Characterization of the agglomerates attached to the tubing lumen

To characterize the agglomerates found on the tubing lumen coming from the pump head region, surface FT-IR spectra (Shimadzu IRAffinity-1S with quest ATR unit, 32 scans) were recorded. Three tubing samples were analyzed: 1) a sample of aged used tubing collected from the pump head region (stored for 3 months at 4°C, Supplementary Figure S6a); 2) a sample of freshly used tubing collected from the pump head region (Supplementary Figure S6b); 3) a sample of new tubing (Supplementary Figure S6c), as reference. The IR data revealed that the agglomerates are silicone debris coming from tubing abrasion caused by continuous cyclic compression.

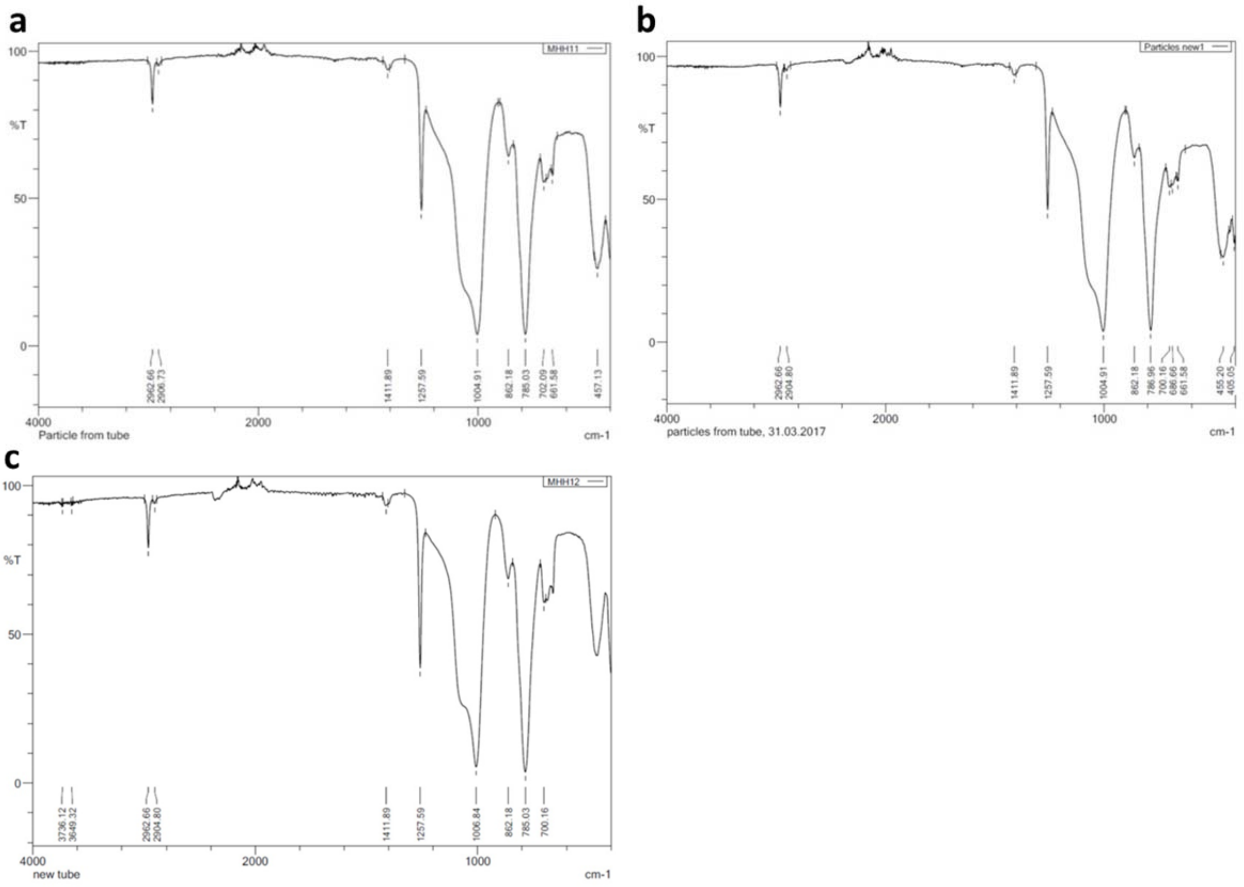

Supplementary Figure S6. Surface FT-IR spectra (ATR). (**a**) FT-IR spectrum of agglomerates found on the lumen of the aged used tubing from the pump head region (storage of 3 months at 4°C). (**b**) FT-IR spectrum of agglomerates found on the lumen of the freshly used tubing from the pump head region (data recorded after 4 days of storage at -20°C). (**c**) FT-IR spectrum of the lumen of the new tubing.

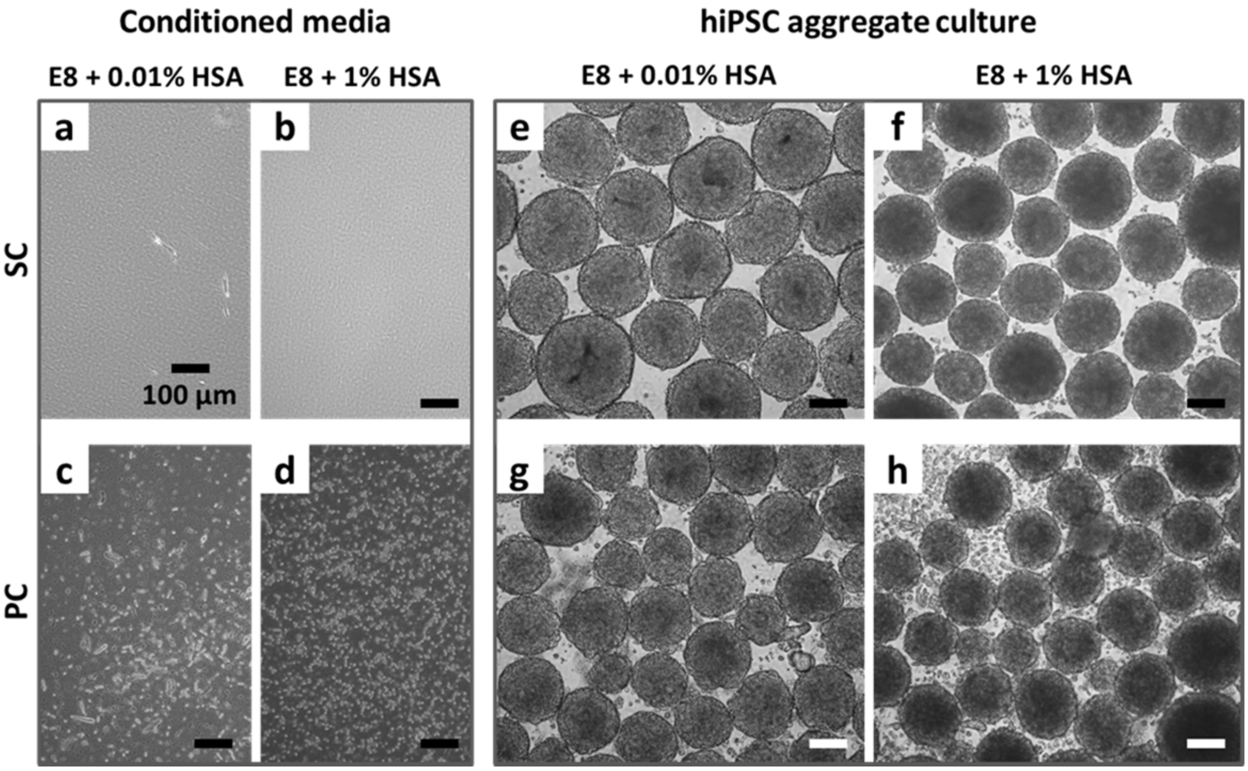

Supplementary Figure S7. Human iPSC aggregates cultured in suspension with SC and PC E8 supplemented with 0.01% HSA or 1% HSA. Light microscopy images of SC E8 supplemented with (**a**) 0.01% HSA and (**b**) 1% HSA showed transparent media. In contrast particle precipitation was observed in both PC E8 supplemented with (**c**) 0.01% HSA and (**d**) 1% HSA. Concerning the hiPSC aggregate culture, (**e**) E8 + 0.01% HSA did not affect the culture when static conditioned but (**g**) showed lower efficacy when peristaltic conditioned, while (**f, h**) the addition of 1% HSA induced detrimental effects for both static and peristaltic conditioning. Scale bar = 100 µm.

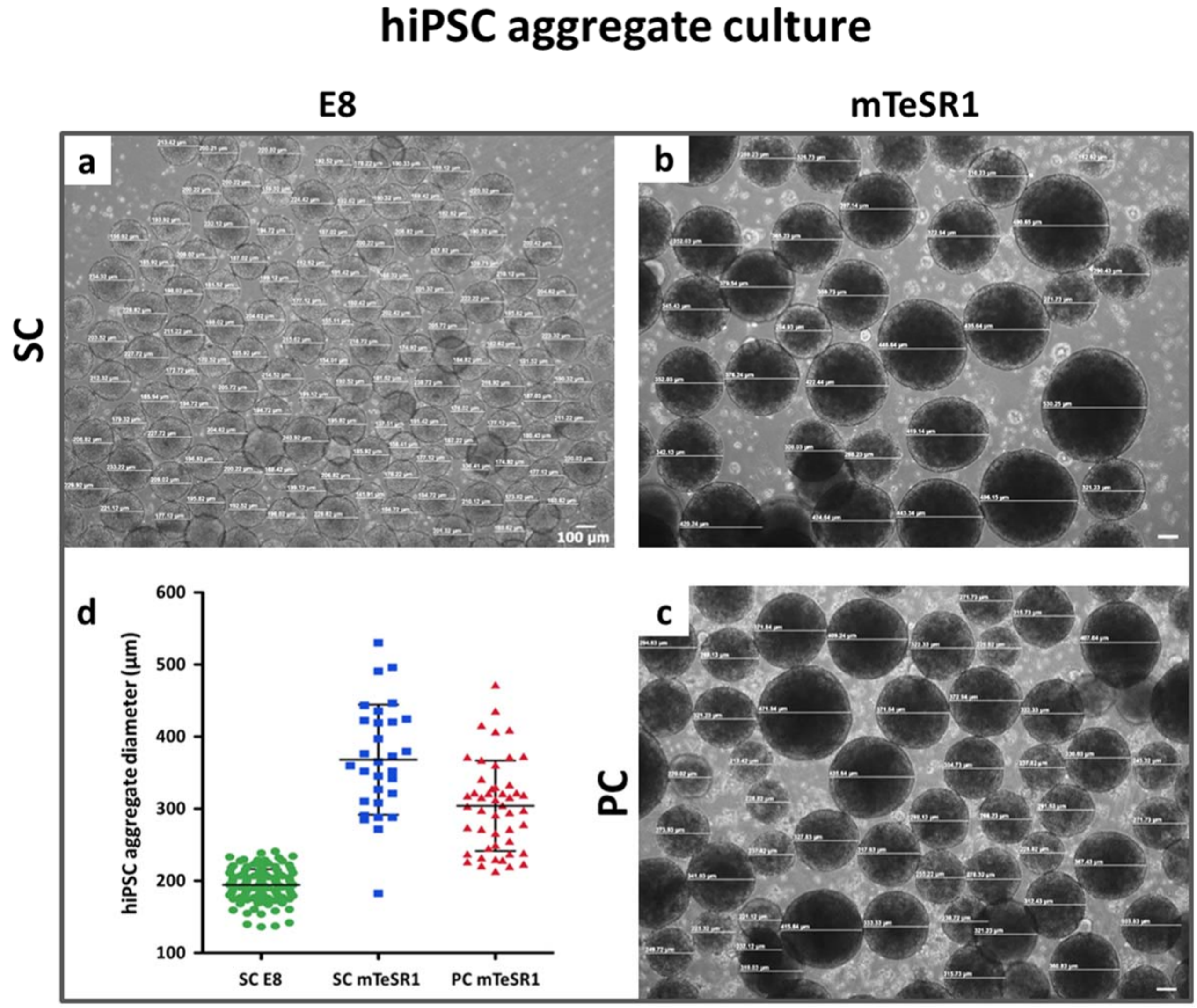

Supplementary Figure S8. Light microscopy images of hiPSC aggregates cultured in (**a**) SC E8 (average diameter = 194.4±21.7 µm), (**b**) SC mTeSR1 (average diameter = 368.1±76.3 µm), and (**c**) PC mTeSR1 (average diameter = 304.1±62.7 µm) with diameter measurement. (**d**) Plot of hiPSC aggregate diameter distribution. Scale bar = 100 µm.

Amount	Component	Supplier
1 L	DMEM/F12, HEPES	Thermo Fisher Scientific Inc., cat. no. 11330057
64 mg/L	Ascorbic acid 2-phosphate	Sigma-Aldrich, cat. no. A8960
14 µg/L	Sodium selenite	Sigma-Aldrich, cat. no. S5261
543 mg/L	NaHCO3	Sigma-Aldrich, cat. no. S6041
20 mg/L	Insulin	Sigma-Aldrich, cat. no. I9278
10.7 mg/L	Human recombinant transferrin	Sigma-Aldrich, cat. no. T3705
100 µg/L	bFGF	Leibniz University Hannover
2 µg/L	TGFβ1 (human recombinant - CHO)	Peprotech, cat. no. 100-21C

Supplementary Table S9. List of components for self-made chemically defined culture medium E8.
